# Genetic Variations in Pattern Recognition Receptor Loci Are Associated with Anti-TNF Response in Patients with Rheumatoid Arthritis

**DOI:** 10.1371/journal.pone.0139781

**Published:** 2015-10-06

**Authors:** Jacob Sode, Ulla Vogel, Steffen Bank, Paal Skytt Andersen, Merete Lund Hetland, Henning Locht, Niels H. H. Heegaard, Vibeke Andersen

**Affiliations:** 1 Department of Autoimmunology and Biomarkers, Statens Serum Institut, Copenhagen, Denmark; 2 Department of Rheumatology, Frederiksberg Hospital, Frederiksberg, Denmark; 3 Institute of Regional Health Research-Center Sønderjylland, University of Southern Denmark, Odense, Denmark; 4 National Research Centre for the Working Environment, Copenhagen, Denmark; 5 Department of Medicine, Viborg Regional Hospital, Viborg, Denmark; 6 Biomedicine, University of Aarhus, Aarhus, Denmark; 7 Department of Microbiology and Infection Control, Statens Serum Institut, Copenhagen, Denmark; 8 Veterinary Disease Biology, University of Copenhagen, Copenhagen, Denmark; 9 The DANBIO Registry, Copenhagen Center for Arthritis Research, Center for Rheumatology and Spine Diseases, Rigshospitalet, Glostrup, Denmark; 10 Department of Clinical Medicine, Faculty of Health and Medical Sciences, University of Copenhagen, Copenhagen, Denmark; 11 Clinical Biochemistry, Clinical Institute, University of Southern Denmark, Odense, Denmark; 12 Molecular Diagnostic and Clinical Research Unit, Hospital of Southern Jutland, Aabenraa, Denmark; 13 OPEN (Odense Patient data Explorative Network), Odense University Hospital, Odense, Denmark; Nippon Medical School Graduate School of Medicine, JAPAN

## Abstract

**Objectives:**

To determine whether genetic variation within genes related to the Toll-like receptor, inflammasome and interferon-γ pathways contributes to the differences in treatment response to tumour necrosis factor inhibitors (anti-TNF) in patients with rheumatoid arthritis (RA).

**Methods:**

In a retrospective case-case study, we assessed 23 functional single nucleotide polymorphisms (SNPs) in 15 genes. We included 538 anti-TNF naïve Danish RA patients from the nationwide DANBIO database. Multivariable logistic regression analyses were performed to detect associations (p-value<0.05) between genotypes and European League Against Rheumatism (EULAR) treatment responses. False Discovery Rate corrections for multiple testing (q-value) and stratified analyses were performed to investigate association with individual therapies and IgM-rheumatoid factor (RF) status.

**Results:**

Six of twenty successfully genotyped polymorphisms were nominally associated with EULAR treatment response. Three of these were in weak to moderate linkage disequilibrium with polymorphisms previously reported associated with anti-TNF treatment response. *TLR5*(rs5744174) variant allele carriers (odds ratio(OR) = 1.7(1.1–2.5),p = 0.010,q = 0.46) and *TLR1*(rs4833095) homozygous variant carriers (OR = 2.8(1.1–7.4),p = 0.037,q = 0.46) had higher odds for a positive treatment response. *NLRP3*(rs10754558) variant allele carriers (odds ratio(OR) = 0.6(0.4–1.0),p = 0.045,q = 0.46) were more likely to have a negative treatment response.

The association in *TLR5*(rs5744174) remained significant after correction for multiple comparisons among patients negative for RF (OR = 6.2(2.4–16.3),p = 0.0002,q = 0.024). No other association withstood correction for multiple testing. Post hoc analyses showed that change in Patient Global score on a visual analogue scale (VAS) and change in pain VAS were the main factors responsible for the association.

**Conclusions:**

We reproduced previously reported associations between genetic variation in the *TLR10/1/6* gene cluster, *TLR5*, and *NLRP3* loci and response to anti-TNF treatment in RA. Changes in VAS pain and patient global scores were the main contributors to the association found for *TLR5*. Furthermore, we identified other candidate genes that require replication in independent cohorts.

## Introduction

Predictive biomarkers capable of stratifying patients into responders and non-responders to treatment with tumour necrosis factor inhibitors (anti-TNF) will enable selection of the optimal treatment for the individual patients and thereby improve patient care. Such biomarkers, however, are scarce and none are applicable in a clinical setting. Some single nucleotide polymorphisms (SNPs) may show associations with anti-TNF response and might be useful for prediction but despite several studies addressing this issue, only few associations have been replicated (e.g. the *PTPRC* [1] and *PDE3A–SLCO1C1* loci [2]). Overall, associations between genetic polymorphisms and anti-TNF response are characterized by small effect sizes.

However, together with e.g. expression profiling, epigenetic, para-clinical, or clinical markers the growing number of polymorphisms associated with anti-TNF treatment response may be potentially useful for prediction [3]. Furthermore, associations between genetic variation and treatment outcome may provide insight into aberrant molecular pathways and thus form the basis for developing new treatment strategies.

We recently published a candidate gene study [4] focussing on genes involved in the NF-κB mediated signalling pathway. Polymorphisms associated with anti-TNF response were found in the genes encoding NACHT, LRR and PYD domains-containing protein 3 (NLRP3/NALP3) (rs4612666) and interferon-γ (IFN-γ) (rs2430561). The association with *NLRP3* is supported by a recent study of British patients [5].

The NLRP3 inflammasome belongs to a group of intracellular innate immune sensors, which generally sense stimuli associated with infection and stressed tissue [6]. Caspase recruitment domain-containing protein 8 (CARD8) is involved in NLRP3-inflammasome formation [6] and polymorphisms in this gene have been associated with response to anti-TNF drugs [5]. Upon inflammasome activation, caspase–1 proteolytically activates the pro-inflammatory cytokines interleukin (IL)-1β and IL–18.

Like the NLRP3-inflammasome, Toll-like receptors (TLRs) are innate immune sensors. They sense exogenous and endogenous antigens and activate pathways (e.g. NF-κB, AP–1) that increase production of pro-inflammatory cytokines. A recent well-powered study found polymorphisms in the TLR signalling pathways (*TLR2*, *TLR4*, *MyD88*, *CHUK*) associated with response to anti-TNF treatment [7]. Also, polymorphisms in the genes encoding TLR–1 and TLR–5 (rs5744174) have been shown to be associated with increased IFN-γ secretion [8,9], and, for the latter, with reduced expression of IL–6 and IL–1β [10].

Interferon-γ (IFN-γ) is a central cytokine for both adaptive and innate immunity that mediates downstream signalling through binding to the heterodimeric IFN-γ receptor (IFNGR1 and IFNGR2), which in turn activates the JAK-STAT (Janus kinase Signal Transducers and Activator of Transcription) pathway [11]. STAT4 regulates proliferation, survival, and differentiation of lymphocytes and genetic variation in the corresponding gene has been associated with RA [12].

Thus, our aim in this study was to assess additional functional polymorphisms related to the TLR, inflammasome and interferon-γ pathways and thereby extend the analysis of associations found in our previous study [4]. We analysed 23 functional polymorphisms in *CARD8*, *IFNGR1*, *IFNGR2*, *IL12B*, *IL12RB1*, *IL12RB2*, *IL17A*, *IL18*, *JAK2*, *NLRP1*, *NLRP3*, *TBX21*, *TIRAP*, *TLR1* and *TLR5* in a Danish cohort of 538 RA patients treated with anti-TNF and prospectively monitored for treatment effect using validated clinical scoring methods.

## Materials and Methods

### Ethics statement

The study was conducted in accordance with the Declaration of Helsinki and was approved by the Regional Ethics Committee of Central Denmark Region (M–20100153 and S–20120113) and the Danish Data Protection Agency (J. 2010-41-4719). The Regional Ethics Committee of Central Denmark Region gave exemption from obtaining informed consent because samples were taken for other reasons and data were analysed anonymously.

### Patients and Samples

We included 538 anti-TNF naïve RA patients. All patients initiated their first anti-TNF treatment, had clinical variables registered at baseline and follow-up in DANBIO (The National Danish Registry for Biological Treatment of Rheumatic Diseases), and had available blood samples. The baseline (pre-treatment) visit was defined as a visit 0–30 days before onset of anti-TNF treatment and follow-up as a visit within 60–180 days after treatment onset (at the contact closest to 120 days if more than one visit was registered). A detailed description of the DANBIO registry and the additional patient data were published previously [4,13].

### Candidate gene analysis

PubMed was searched for functional polymorphisms in genes closely related to the up- and down-stream signalling molecules of the NLRP3 inflammasome and interferon-γ [4]. A total of 23 SNPs were ultimately chosen primarily based on evidence of biological effect and secondly based on documented association with autoimmune disease (S1 Table). Expected minor allele frequencies (MAFs) ranged from 14% to 44% except for one polymorphism (rs11810249, MAF: 4.4%).

The polymorphisms were genotyped by PCR-based KASP genotyping assay by LGC Genomics (Middlesex, United Kingdom—www.lgcgenomics.com) on DNA extracted from blood (Maxwell 16 LEV Blood DNA Kit, Promega, Madison, Wisconsin, USA) as described by Bank et al. [14]. Genotyping failed for rs8134145 (*IFNGR2*) and rs2072493 (*TLR5*) due to their close proximity to neighbouring genotyped SNPs, and for *IL17A* for unknown reasons. All other chosen assays had a call rate exceeding 95%. Repeated genotyping of 94 randomly selected samples in an inflammatory bowel disease cohort yielded >99% identical calls.

### Statistical methods

Primary outcome (good, moderate or none) was defined by the European League Against Rheumatism (EULAR) response criteria [15] at follow-up. We performed the analyses by comparing either EULAR good/moderate vs. none (G/M vs. N) response or EULAR good vs. moderate/none (G vs. M/N) response. We also analysed treatment response defined by the American College of Rheumatology outcome measure, ACR50 response [16] and relative change in disease activity score for 28 joints (DAS28) (*relDAS28 = (baseline DAS28 − follow-up DAS28)/baseline DAS28*) to enable comparison with other studies.

Multivariate logistic regression analyses were performed to assess association between genotype and anti-TNF treatment response at the 5% significance level. Adjustments were made in the multivariate analyses for sex, baseline health assessment questionnaire (HAQ) score, DAS28 and concomitant treatment with disease modifying anti-rheumatic drug (DMARD). We have also performed stratified analyses for patients positive for IgM-RF (seropositive RA patients), for the specific type of anti-TNF drug (not certolizumab and golimumab due to insufficient power) and for a combined group of patients treated with monoclonal antibodies (infliximab, adalimumab, and golimumab).

We performed correction for multiple testing using False Discovery Rate classical one-stage method set at 0.05 (q-value) [17]. Values were based on the number of test performed in each of the primary, secondary and stratified analyses, respectively (see S1–S6 tables). Values can be interpreted as the expected proportion of false positive results at least as extreme as the observed result.

## Results

### Study population

Baseline characteristics and treatment response of the study population are presented in Table 1. Seronegative RA patients were significantly younger and had higher pain and global scores as well as higher tender joint count compared with the seropositive patients. No difference in response rates to anti-TNF was observed between the two groups. Both had 29–30% non-responders. Patients with RA receiving concomitant synthetic DMARDs (sDMARD) comprised 84%.

**Table 1:** Baseline clinical characteristics and treatment response of the study population.

	All RA patients	Seropositive RA patients	Seronegative RA patients	P-value #
Number	538	407 (75.7%)	131 (24.3%)	
Female	407 (75.7%)	302 (74.2%)	105 (80.2%)	0.17
Mean age / years (SD)				
at treatment start	55.0 (13.0)	55.7 (12.8)	53.0 (13.6)	0.04
Smoking status				
Current	142 (31.8%)	110 (32.7%)	32 (29.1%)	0.56
Previous	151 (33.9%)	120 (35.7%)	31 (28.2%)	0.20
Never	153 (34.3%)	106 (31.6%)	47 (42.7%)	0.03
Missing data	92 (-)	71 (-)	21 (-)	0.71
Synthetic DMARD at baseline	452 (84.0%)	342 (84.0%)	110 (84.0%)	0.99
Number of synthetic DMARDs (1/2/3)	61.7%/16.4%/5.9%	61.7%/16.0%/6.4%	61.8%/17.6%/4.6%	ns.
Methotrexate	396 (73.6%)	299 (73.5%)	97 (74.0%)	0.90
Salazopyrine	120 (22.3%)	92 (22.6%)	28 (21.4%)	0.77
Chloroquine	70 (13.0%)	55 (13.5%)	15 (11.5%)	0.54
Leflunomide	10 (1.9%)	7 (1.7%)	3 (2.3%	0.67
Azathioprine	8 (1.5%)	6 (1.5%)	2 (1.5%)	0.97
Erosive status				
Erosions	308 (65.4%)	258 (70.9%)	50 (46.7%)	<0.0001
Missing data	67 (-)	43 (-)	24 (-)	
Baseline patient global score (VAS 0–100) / Mean (SD)	62.6 (22.6)	60.8 (22.9)	68.5 (20.7)	0.0006
Δ patient global score / Mean (SD)	22.1 (28.1)	21.6 (1.4)	23.7 (29.5)	0.45
Baseline physician global score (VAS 0–100) / Mean (SD)	38.4 (20.7)	38.0 (20.2)	39.8 (22.3)	0.38
Baseline pain score (VAS 0–100) / Mean (SD)	58.0 (22.8)	55.9 (23.2)	64.4 (20.2)	0.0002
Δ pain score / Mean (SD)	21.4 (28.0)	20.9 (28.0)	22.9 (28.0)	0.48
TJC 0–28 / Mean (SD)	9.5 (7.3)	9.0 (7.0)	11.1 (8.1)	0.004
SJC 0–28 / Mean (SD)	5.4 (4.6)	5.6 (4.5)	4.8 (4.8)	0.08
HAQ score (VAS 0–100) / Mean (SD)	1.2 (0.7)	1.2 (0.7)	1.3 (0.7)	0.72
CRP / mg/mL (SD)	19.7 (25.5)	20.5 (27.0)	17.2 (20.3)	0.20
DAS28 / mean (SD)	4.9 (1.2)	4.8 (1.2)	5.0 (1.1)	0.14
ΔDAS28 / mean (SD)	1.5 (1.4)	1.5 (1.5)	1.5 (1.4)	0.81
Anti-TNF drug				
Infliximab (%)	168 (31.2%)	122 (30.0%)	46 (35.1%)	0.27
Etanercept (%)	166 (30.8%)	124 (30.5%)	42 (32.1%)	0.73
Adalimumab (%)	134 (24.9%)	105 (25.8%)	29 (22.1%)	0.40
Golimumab (%)	49 (9.1%)	38 (9.3%)	11 (8.4%)	0.75
Certolizumab (%)	21 (3.9%)	18 (4.4%)	3 (2.3%)	0.27
EULAR response				
Good (%)	231 (42.9%)	178 (43.7%)	53 (40.5%)	0.51
Moderate (%)	148 (27.5%)	108 (26.5%)	40 (30.5%)	0.37
None (%)	159 (29.6%)	121 (29.7%)	38 (29.0%)	0.87
ACR50 response (%)	170 (31.6%)	131 (32.2%)	39 (29.8%)	0.61
RelDAS28 response (SD)	0.28 (0.32)	0.28 (0.34)	0.28 (0.27)	0.91

SD: standard deviation; DMARD: disease modifying anti-rheumatic drugs; VAS: visual analogue scale; ΔVAS: baseline VAS minus follow-up VAS; TJC: tender joint count; SJC: swollen joint count; HAQ: health assessment questionnaire; CRP: C-reactive protein; DAS28: disease activity score (28-joints); EULAR: European League Against Rheumatism; ACR50: American College of Rheumatology, 50% improvement; RelDAS28: relative change in DAS28;

^#^: Two-sided t-test p-value of difference in means/proportions between seropositive and seronegative patients; Seropositive: Positive for IgM-RF

Clinical data were collected at variable time-points due to the study design. Eighty-seven per cent of the patients had baseline data collected 7 days or less before treatment onset, and 74% of the follow-up data were registered 16±4 weeks after treatment onset (S1 Fig).

### Genotype associations with anti-TNF response

We assessed the association with EULAR anti-TNF response regardless of RF status among all patients. We found that variant allele carriers of the *TLR5* rs5744174 (OR = 1.7, p = 0.010), *IL18* rs187238 (OR = 1.5, p = 0.026) and the homozygous variant genotype of *TLR1* rs4833095 (OR = 2.8, p = 0.037) had a positive treatment response (EULAR G vs. M/N) (Table 2). With EULAR G/M vs. N as treatment response, carriers of another variant in *IL18* (rs1946518; r^2^ = 0.59) were more likely to have positive treatment response (OR = 1.6, p = 0.022). Furthermore, individuals heterozygous for *IL12B* rs6887695 (OR = 0.6, p = 0.017) and variant allele carriers of *NLRP3* rs10754558 (OR = 0.6, p = 0.017) seemed to have a less favourable treatment response (EULAR G/M vs. N).

**Table 2:** Adjusted odds ratios for associations between genotypes and EULAR anti-TNF treatment response.

				EULAR	
				G/M vs. N	G vs. M/N
Gene	Genotype	Freq.	G/M/N	OR (95% CI), p-, q-value	OR (95% CI), p-, q-value
SNP					
IL12B	GG	241	109/70/62		
rs6887695	GC	224	91/54/79	**0.60 (0.40–0.91), 0.017** *, **0.46**	0.82 (0.56–1.20), 0.31, 0.70
	CC	51	20/22/9	1.44 (0.64–3.22), 0.38, 0.72	0.81 (0.43–1.53), 0.52, 0.74
	GC/CC	275	111/76/88	0.69 (0.46–1.03), 0.068, 0.47	0.82 (0.57–1.17), 0.28, 0.65
IL18	GG	254	96/76/82		
rs187238	GC	198	91/51/56	1.39 (0.91–2.13), 0.13, 0.48	**1.50 (1.02–2.21), 0.041** *, **0.46**
	CC	55	26/16/13	1.85 (0.91–3.74), 0.089, 0.47	1.58 (0.86–2.90), 0.14, 0.48
	GC/CC	253	117/67/69	1.48 (0.99–2.21), 0.057, 0.47	**1.52 (1.05–2.19), 0.026** *, **0.46**
IL18	GG	187	73/50/64		
rs1946518	GT	246	111/70/65	**1.57 (1.02–2.43), 0.041** *, **0.46**	1.36 (0.92–2.03), 0.13, 0.48
	TT	82	36/24/22	1.72 (0.94–3.16), 0.077, 0.47	1.25 (0.73–2.16), 0.42, 0.72
	GT/TT	328	147/94/87	**1.61 (1.07–2.42), 0.022** *, **0.46**	1.34 (0.92–1.94), 0.13, 0.48
NLRP3	CC	181	86/50/45		
rs10754558	CG	243	96/69/78	**0.61 (0.39–0.96), 0.033** *, **0.46**	0.71 (0.48–1.06), 0.098, 0.48
	GG	85	35/24/26	0.75 (0.41–1.36), 0.34, 0.71	0.80 (0.47–1.37), 0.42, 0.72
	CG/GG	328	131/93/104	**0.64 (0.42–0.99), 0.045** *, **0.46**	0.74 (0.50–1.07), 0.11, 0.48
TLR1	TT	312	130/91/91		
rs4833095	TC	178	75/53/50	1.17 (0.76–1.80), 0.47, 0.72	1.06 (0.72–1.55), 0.78, 0.91
	CC	21	14/2/5	1.09 (0.37–3.20), 0.87, 0.92	**2.80 (1.07–7.35), 0.037** *, **0.46**
	TC/CC	199	89/55/55	1.16 (0.77–1.76), 0.48, 0.72	1.17 (0.81–1.69), 0.41, 0.72
TLR5	TT	170	61/55/54		
rs5744174	TC	234	104/62/68	1.25 (0.80–1.96), 0.33, 0.71	**1.55 (1.02–2.35), 0.040** *, **0.46**
	CC	107	53/26/28	1.51 (0.86–2.66), 0.16, 0.50	**1.96 (1.18–3.25), 0.009** **, **0.46**
	TC/CC	341	157/88/96	1.32 (0.87–2.02), 0.19, 0.57	**1.67 (1.13–2.46), 0.010** *, **0.46**

Logistic regression, adjusted for gender, HAQ, DMARD, and disease activity score (28-joints) at baseline; CI: confidence interval; Freq.: Frequency; OR: odds ratio; EULAR, G/M/N: European League Against Rheumatism response criteria, good/moderate/none; P-value:

*<0.05,

**<0.01;

q-value: False Discovery Rate classical one-stage method set at 0.05.

Analyses of associations with treatment response outcome defined by ACR50 and relDAS28 are shown in S2 Table. Association with altered ACR50 treatment response was found for *TLR1* rs4833095 and with altered relDAS28 treatment responses for *NLRP3* rs10754558 and *TLR5* rs5744174.

We also assessed the functionally relevant haplotypes in *IFNGR2* and *IL12B* (S1 Table) for association with EULAR anti-TNF response (G/M vs. N and G vs. M/N) under the same multivariate model. No associations between risk-haplotypes and anti-TNF response and no interaction with smoking were found.

#### Stratified analyses

Next, we performed stratified analyses of polymorphisms for associations with treatment response.

For seropositive RA patients (n = 407), *IL12B* rs6887695, *IL18* rs187238 and *NLRP3* rs10754558 were associated with similar effect estimates and significance levels (S3 Table) as the total cohort. For *IL18* rs1946518 and *TLR1* rs4833095 effect estimates were similar (p-value <0.1) and for *TLR5* rs5744174 effect estimates were substantially lower and non-significant compared to the overall cohort (S3 Table).

Stratified analyses by type of anti-TNF drug (infliximab (n = 168), etanercept (n = 166), adalimumab (n = 134) and the combined group of monoclonal antibodies (infliximab, adalimumab, and golimumab) (n = 372)) are shown in S4 Table. Variant allele carriers of *TLR5* rs5744174 treated with infliximab (OR = 2.7, p = 0.018) and etanercept (OR = 2.3, p = 0.022) were more likely to have a good treatment response (EULAR G vs. MN) (S4 Table) and a clear allele-dosage effect was observed. Altered treatment responses were also seen among variant allele carriers of *NLRP1* (rs878329) and *NLRP3* rs10754558 treated with infliximab and among variant allele carriers of *IL12B* rs6887695 treated with the monoclonal anti-TNF drugs (S4 Table).

#### Post hoc analyses

**Fig 1:**
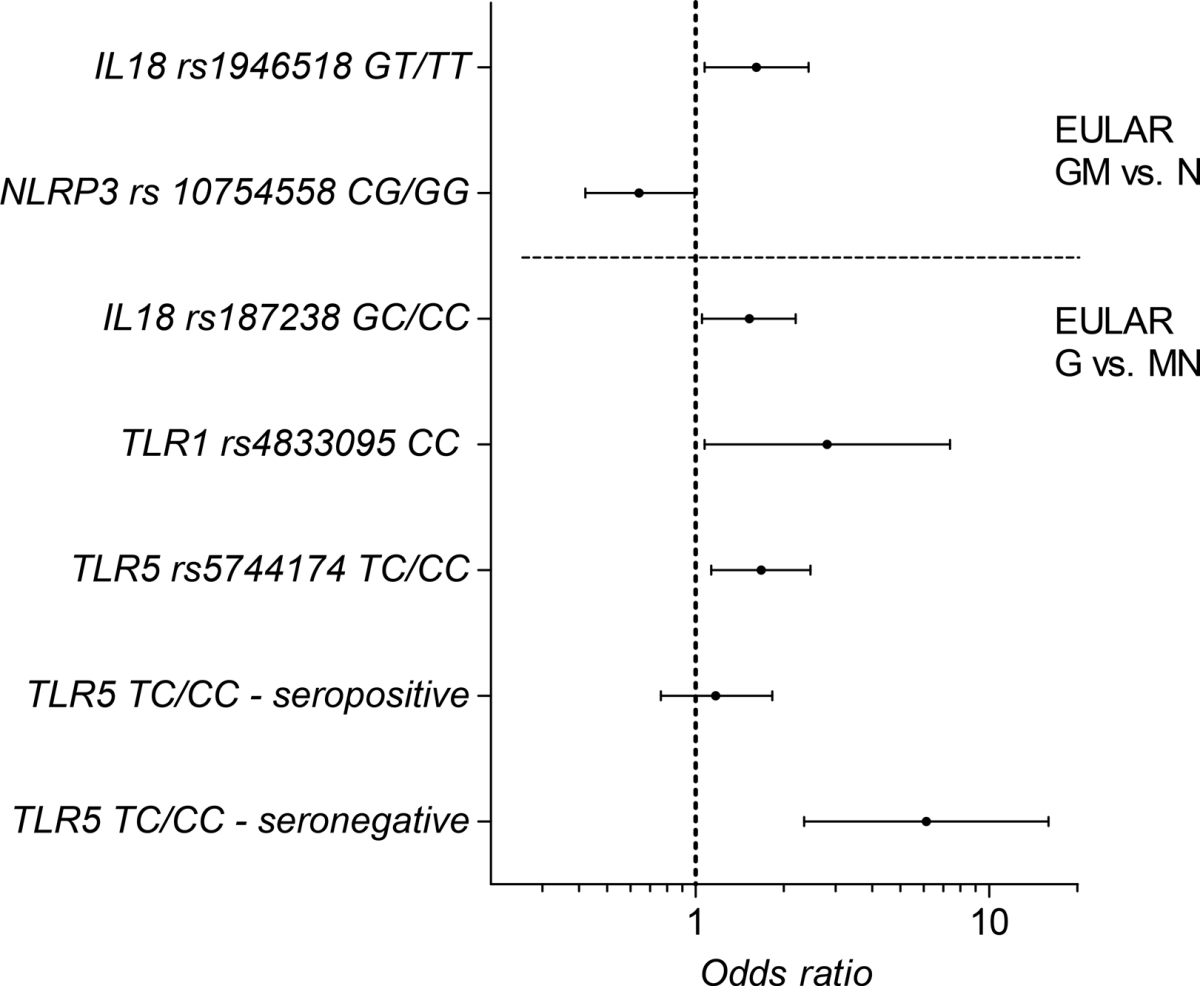
Odds ratio for association between polymorphisms in *IL18*, *NLRP3*, *TLR1* and *TLR5* and EULAR good/moderate vs. none and good vs. moderate/non-response, respectively. For *TLR5* rs5744174, patients were also stratified on diagnosis based on IgM-RF (seropositive-/seronegative RA). Log scale, 95% confidence interval.

Since there was some discrepancy between associations found in the primary analyses and stratified analyses of seropositive patients, we also assessed the association in the seronegative subgroup of RA patients for *TLR5* rs5744174 (S3 Table). Seronegative carriers of the variant allele of *TLR5* rs5744174 had a greater chance of good treatment response (EULAR G vs. M/N) (OR = 6.2 (2.4–16.3), p = 0.0002, q = 0.024) (Fig 1).

Due to lack of association among seropositive RA patients with the *TLR5* polymorphism, we explored other clinical variables of importance for this association using linear regression analyses of the association between the *TLR5* polymorphism and the components of DAS28 (change (Δ) between baseline and follow-up of swollen and tender joint counts (SJC and TJC), CRP and patient global score).

Interestingly, *TLR5* rs5744174 was strongly associated with Δ patient global score but neither with Δ SJC (p = 0.66), Δ TJC (p = 0.20) nor with Δ CRP (p = 0.22). Using linear regression the association remained stable both in a crude analysis (regression coefficient (RC) = 7.8 (2.7–12.8), p-value = 0.003) and in a multivariate model adjusting for baseline patient global score plus the same variables as in the primary analyses (RC = 7.4 (2.6–12.1), p = 0.002). Almost identical results were found when we substituted Δ patient global score with Δ pain score in a similar analysis (RC = 6.8 (95% CI: 2.2–11.4), p = 0.004).

In this cohort of RA patients, there were marked clinical differences between the seropositive and seronegative subgroups (fraction of smokers, erosive status, age, baseline tender joint count, pain and patient global score), and this could potentially cause confounding. However, additional multivariate regression analysis adjusting for these potential confounders did not markedly change the significance levels or effect sizes for the RF status-stratified analyses of *CARD8* (rs2043211), *IFNGR2* (rs17882748), *IL12B* (rs6887695), *IL18* (rs187238, rs360719), *TLR5* (rs5744174) or *TLR1* (rs4833095) (S6 Table).

## Discussion

In this study, we extended the analyses of polymorphisms in genes related to inflammasome- and interferon-γ pathways. Overall, we successfully genotyped 20 of 23 functional polymorphisms, and six SNPs in *IL12B*, *IL18*, *NLRP3*, *TLR1* and *TLR5* (Table 2) were nominally associated with EULAR treatment response in RA patients treated with anti-TNF. Furthermore, the *TLR5* polymorphism lacked association among the seropositive patients but was strongly associated with response among the seronegative patients.

We chose to analyse at the 0.05 significance level. At this level, we had ≥80% chance to detect effect sizes between 1.3 and 1.5 assuming an additive genetic model, and effect sizes between 1.6 and 2.9 assuming a recessive model (MAF between 0.1–0.4). Since multiple testing increases the risk of false positive associations, we have also presented associations corrected for multiple testing (False Discovery Rate, q-value). The association found for *TLR5* among seronegative RA patients was the only that withstood correction for multiple testing.

Since the present study is exploratory, replication in independent cohorts is needed, in particular, for those found in the stratified analyses. However, the selection of functional polymorphisms in genes with a biologically plausible role in RA pathogenesis increases the prior probability of true associations. The presented work does not provide evidence for causal relationships between the studied polymorphisms and the altered anti-TNF response because the associated polymorphisms could also be proxies for other genetic markers.

It should be noted that, due to the design of the study, the time-points of collected response data vary between 60 and 180 days from treatment onset. When we analysed the subset of patients with response data at 16±4 weeks (comprising 74% of the patients) in the overall cohort, associations found for *IL12B* r**s6**887695, *IL18* rs1946518, and the SNPs in *TLR1* and *TLR5* remained nominally associated with similar or higher odds ratios (S5 Table).

The contrasting odds ratios for *IL12B* rs6887695 heterozygous and homozygous variant genotypes substantially lower the likelihood of a true association for this polymorphism.

**Fig 2:**
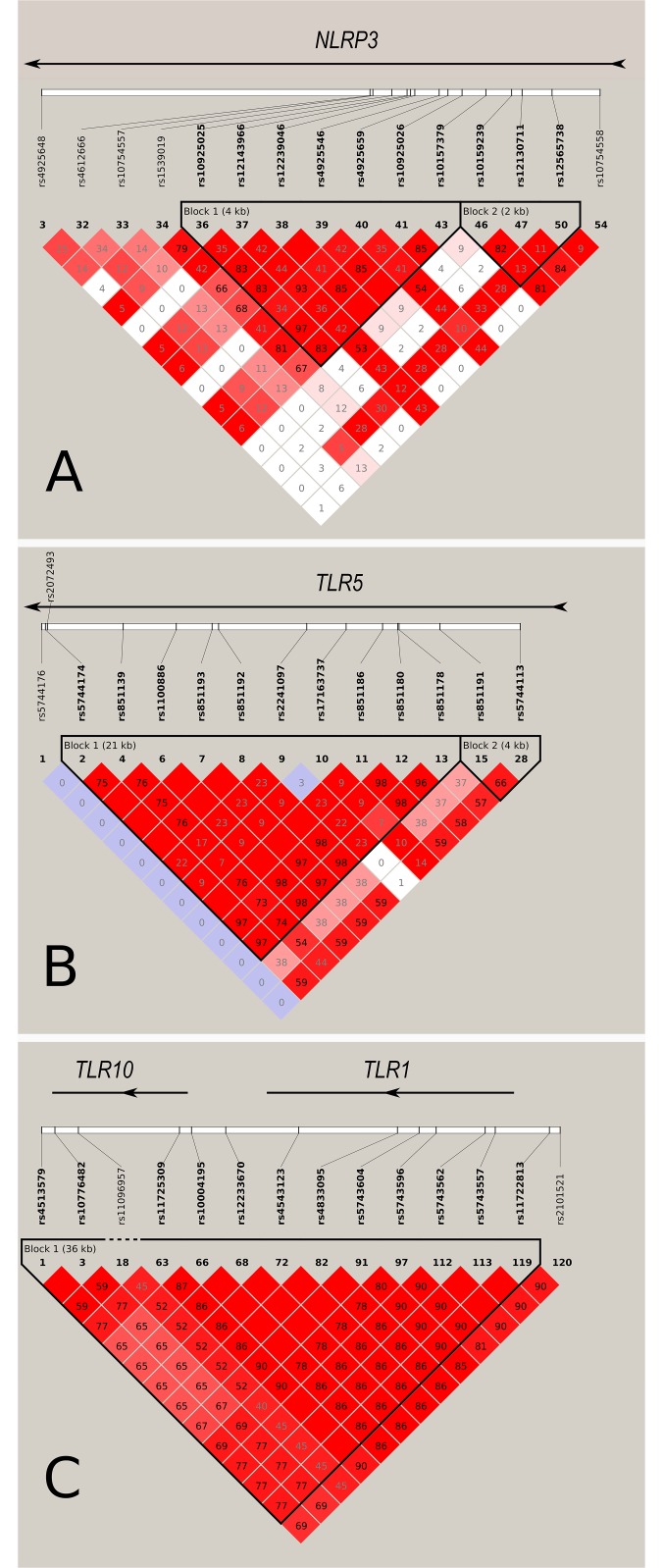
Linkage disequilibrium-maps for A) *NLRP3*, B) *TLR5*, and C) *TLR10/1*. Numbers in squares represent r^2^. Darker red indicates stronger linkage disequilibrium. Maps were made using Haploview software version 4.2 and CEPH/CEU HapMap dataset (Phase II+III merged, release 28/ August10). HapMap data were downloaded by respective genes (*TLR5* and *NLRP3*) and for *TLR10/1* data spanning both genes. To simplify LD-maps, SNPs were selected in the following way: NLRP3: minor allele frequency (MAF) >0.1, Hardy-Weinberg equilibrium (HW) p-value >0.01, genotype>50%, force include: rs10754558, force exclude #4–29; TLR5: MAF >0.1, HW p-value >0.01, genotype>50%, force include rs5744176, force exclude # 17–25; TLR10/1: MAF >0.1, HW p-value >0.01, genotype>50%, force include rs11096957, force exclude #4–58, 98–111.

The present study reproduces associations between three gene loci (*TLR10/1/6* gene cluster, *NLRP3* and *TLR5*) (Fig 2) and RA anti-TNF response previously found in independent studies [4,5,7,18]. *TLR1* rs4833095 is located within a cluster of genes encoding TLR–1, TLR–6 and TLR–10 in a 54-kb region on chromosome 4. *TLR1* rs4833095 is in modest linkage disequilibrium with *TLR10* rs11096957 (R^2^ = 0.52, D’ = 0.94), which was found associated with EULAR GM vs. N response to anti-TNF treatment in a UK cohort (N = 909) [7]. *TLR1* rs4833095 has been associated with a high peripheral blood mononuclear cell (PBMC) TLR–1 cell surface expression [19]. Here, we found the homozygous variant genotype associated with a greater chance of good treatment response to anti-TNF. However, this finding relies on small patient groups and needs confirmation in an independent and preferably larger cohort.

To our knowledge, this study is the third to show an association between genetic variation in *NLRP3* and anti-TNF treatment response. Mathews et al. found three polymorphisms in *NLRP3* with nominal association with EULAR response with rs4925659 as the most tightly linked with rs10754558 (D’ = 0.93; r^2^ = 0.40) [5]. In the same cohort as used in the present study, we have previously reported the rs4612666 T allele to be associated with a lesser chance of EULAR GM vs. N response [4]. The two SNPs are not in linkage disequilibrium (D’ = 0.15; r^2^ = 0.06), thus not allowing haplotype analysis.

Both rs4612666 and rs10754558 in *NLRP3* have been functionally investigated by Hitomi et al. [20]. In THP–1 cells they found minor alleles of these two polymorphisms to decrease and increase *NLRP3* expression, respectively. Based on this, our results for these two polymorphisms seem contradictory since the high expression alleles of the two polymorphisms are associated with positive and negative treatment response, respectively. However, the in vitro experiments performed on THP–1 cells may not reflect in vivo conditions in RA patients.

We found two polymorphisms in *IL18* associated with anti-TNF treatment response. IL–18 is an interleukin that is activated by the NLRP3 inflammasome, and these results therefore further support a role of this pathway for anti-TNF treatment outcome in RA.

The association found for *TLR5* confirms the association between a *TLR5* gene locus and EULAR anti-TNF response previously found in a Dutch cohort (n = 182) [18]. In that study, both rs5744174 and another *TLR5* polymorphism (rs2072493) in linkage disequilibrium (genetic distance: 71bp; D’ = 1; r^2^ = 0.08) were genotyped (Fig 2). Only rs2072493 was associated with anti-TNF response. In a Swedish validation cohort (n = 269), these findings could not be confirmed. Due to different MAFs of these two polymorphisms, they have low tagging capabilities of each other (r^2^ = 0.08). The lack of associations for rs5744174 in the Dutch cohort and for rs2072493 in the Swedish cohort could be caused by the low statistical power, causing false negative results. Differences in statistical methods may also play a role. Coenen et al. performed unadjusted Fisher’s exact or Chi-square test for analyses of 3x3 tables (EULAR response groups / genotype counts), and analyses of our data are non-significant when analysed this way (data not shown). Further, differences in anti-TNF drugs received in the two cohorts may also be of importance. As an example, the fraction of adalimumab-treated patients is larger in the Dutch cohort (34% vs. 25%). Our data do suggest a trend for a negative adalimumab response for carriers of the *TLR5* variant allele compared to a positive treatment response found in the overall cohort and among infliximab and etanercept treated patients (S4 Table).

Functional studies of *TLR5* rs5744174 have shown that flagellin (a TLR5 ligand) stimulation results in lower chemokine (C-C motif) ligand (CCL20) production in HEK 293T cells [21] and reduced IL–6 and IL–1β expression in primary immune cells from healthy homozygous carriers of the variant allele [10]. Another study reported that anti-TNF treatment decreases TLR5 (and TLR4) expression among ankylosing spondylitis patients [22], which supports the hypothesis of TLR5 having a role in the differential anti-TNF response.

Interestingly, post hoc analyses showed that change in patients’ subjective general health assessment (Δ patient global score) and probably also change in pain score could explain most of the correlation with the multi-component EULAR response. A recent paper by Coenen et al. showed that separate components of DAS28, such as tender and swollen joint counts, have a stronger heritability in pharmacogenetic studies of anti-TNF response than DAS28 itself [23]. Although they did not show this for patient global score assessment, it may be the case for *TLR5* rs5744174. In fact, it is well established that TLRs are central in chronic pain pathogenesis [24] and TLR2/3/4/5 deficient mice have reduced neuropathic pain [25]. In a future validation study of the predictive value of this polymorphism, it will therefore be very relevant to include analyses adjusting for patient global score and RF status.

Overall, the effects of the associated polymorphisms were too weak to be used as independent biomarkers for treatment response but they have a potential use as part of a larger panel of predictive biomarkers if validated in independent cohorts.

Based on the results from this study, we hypothesize that so far undefined RA sub-phenotype(s) not responding to anti-TNF treatment are characterized by increased NLRP3-inflammasome activation and increased IL–1β/IL–18 production.

In conclusion, we have reproduced previously published associations between genetic variation in the *TLR10/1/6* gene cluster, *TLR5*, and *NLRP3* loci and response to anti-TNF treatment in RA. Changes in VAS pain and patient global scores were main factors in the association found for *TLR5*. SNPs in *IL18* were associated with anti-TNF treatment response, but these associations need validation in an independent cohort.

## Supporting Information

S1 Fig Registered response data. Quantile-plot of distribution of treatment duration at time of response classification.
(TIF)

S1 Table Chosen polymorphisms and corresponding gene. Associated effect of polymorphism.
(DOCX)

S2 Table ACR50 and relDAS28 anti-TNF treatment response. Adjusted odds ratio (OR)/coefficient for associations between genotypes and ACR50 and relDAS28 response to anti-TNF treatment. (a. All RA patients, b. Seropositive RA patients, c. Seronegative RA patient for *TLR5* rs5744174).
(DOCX)

S3 Table EULAR anti-TNF treatment response. Adjusted odds ratios for associations between genotypes and EULAR anti-TNF treatment response. (a. All RA patients, b. Seropositive RA patients, c. Seronegative RA patient for *TLR5* rs5744174).
(DOCX)

S4 Table Anti-tumour necrosis factor (TNF) drug stratified analyses. Odds ratio for variant allele carriers association with EULAR good vs. moderate/none response. (a. EULAR good/moderate vs. non-response. b. EULAR good vs. moderate/non-response).
(DOCX)

S5 Table EULAR anti-TNF treatment response at 16 weeks. All RA patients. Adjusted odds ratios for associations between genotypes and EULAR anti-TNF treatment response.
(DOCX)

S6 Table IgM-Rheumatoid factor stratified analyses adjusted for potential confounders. Multivariate logistic regression. Odds ratio (OR) per variable in model for European League Against Rheumatism response criteria, good/moderate/none (EULAR, G/M/N). Seropositive/-negative RA: rheumatoid arthritis positive/negative for IgM-rheumatoid factor. DAS28: disease activity score across 28-joints. HAQ: health assessment questionnaire score. DMARD: disease-modifying anti-rheumatic drugs (dichotome). Smoking: previous/current smoking (dichotome). Erosions: erosions on x-ray (dichotome). TJC: tender joint count. VAS: visual analogue scale. P-value: * <0.05. ** <0.01. *** <0.001.
(DOCX)

## Abbreviations

ACR50 response: American College of Rheumatology outcome measure, 50% improvement
EULAR G/M/N: European League Against Rheumatism good/moderate/none response
HAQ: health assessment questionnaire
MAF: minor allele frequency
relDAS28: relative change in disease activity score for 28 joints
SJC: swollen joint count
TJC: tender joint count
