# Supplementary figures and images for: Genetic Variations in Pattern Recognition Receptor Loci Are Associated with Anti-TNF Response in Patients with Rheumatoid Arthritis
Source: PLoS One. 2015 Oct 6;10(10):e0139781. doi: 10.1371/journal.pone.0139781 (PMC4595012; doi:10.1371/journal.pone.0139781)

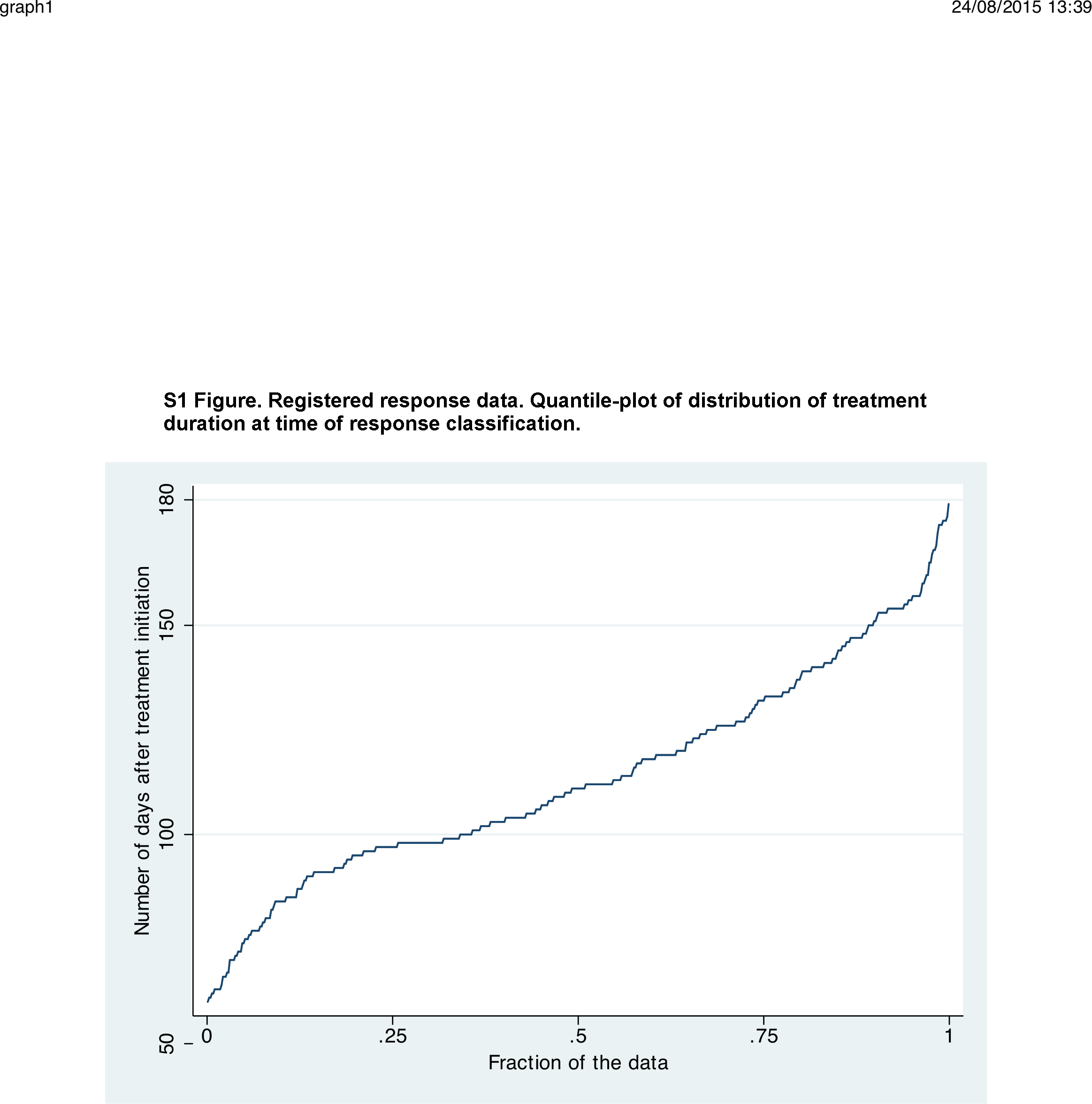

Supplement: S1 Fig — Quantile-plot of distribution of treatment duration at time of response classification. (TIF) [file pone.0139781.s001.tif]
