# Supplementary material for: Genetic Variations in Pattern Recognition Receptor Loci Are Associated with Anti-TNF Response in Patients with Rheumatoid Arthritis
Source: PLoS One. 2015 Oct 6;10(10):e0139781. doi: 10.1371/journal.pone.0139781 (PMC4595012; doi:10.1371/journal.pone.0139781)
Supplement: S1 Table — Associated effect of polymorphism. (DOCX) [file pone.0139781.s002.docx]

**S1 Table. Chosen polymorphisms and corresponding gene.** Associated effect of polymorphism.

| GENE | GENETIC VARIANT | WILDTYPE/ VARIANT | NCBI GENE ID | MAF | PROTEIN FUNCTION | ASSOCIATED EFFECT OF POLYMORPHISM |
| --- | --- | --- | --- | --- | --- | --- |
| *CARD8* | rs2043211 | A/T | 22900 | 0.274 | Caspase recruitment domain-containing protein 8 is part of inflammasome formation. See *NLRP3*. | T allele decreases expression^F^ [1] and is associated with worse disease course in early RA [2]. |
| *IFNGR1* | rs2234711 | C/T | 3459 | 0.353 | IFN-γ receptor is a heterodimer of IFNGR1 and IFNGR2. Ligand binding activates the JAK-STAT pathway. | T allele increases expression^F^ [3]. |
| *IFNGR2* | rs8134145 | C/A | 3460 | 0.306^A, B^ | See *IFNGR1*. | *IFNGR2* ATC haplotype increases transcription^F^ [4]. |
| *IFNGR2* | rs8126756 | T/C | 3460 | 0.308^A^ | See *IFNGR1*. | *IFNGR2* ATC haplotype increases transcription^F^ [4]. |
| *IFNGR2* | rs17882748 | T/C | 3460 | 0.414^A^ | See *IFNGR1*. | *IFNGR2* ATC haplotype increases transcription^F^ [4]. |
| *IL12B* | rs3212217 | G/C | 3593 | 0.190 | IL-12-p40 serves as a subunit of interleukin 12 and 23. IL-12 can activate IFN-γ signalling pathway. | C allele increases IL-12 Secretion Levels by PBMCs In Vitro^G^ [5]. |
| *IL12B* | rs3212227 | A/C | 3593 | 0.190 | IL-12 binding to the IL-12 receptor activates JAK2 and tyrosine kinase 2 (TYK2) and leads to phosphorylation of STAT4. | C allele associated with decreased IL-12p40 level^G^ [6–8] Psoriasis risk allele [9]. |
| *IL12B* | rs6887695 | G/C | 3593 | 0.326 | See above. | C associated with decreased IL-12p40 level in haplotype context with *IL12B* rs3212227^G^ [8]. Psoriasis risk allele [9]. |
| *IL12RB1* | rs401502 | C/G | 3594 | 0.375 | Subunit of IL-12 receptor and IL-23 receptor. IL-12 binding to the IL-12 receptor activates JAK2 and tyrosine kinase 2 (TYK2) and leads to phosphorylation of STAT4. | G allele reduce IL-12p40 level^E^ [6]. |
| *IL12RB2* | rs11810249 | C/T | 3595 | 0.044^A^ | Subunit of IL-12 receptor. IL-12 binding to the IL-12 receptor activates JAK2 and tyrosine kinase 2 (TYK2) and leads to phosphorylation of STAT4. | T allele reduces transcription^D, F^ [10]. |
| *IL17A* | rs8193036 | T/C | 3605 | 0.241^B^ | Induces cytokine production in various cell-types. | T allele increases expression^C, D^. Associated with UC [11]. |
| *IL18* | rs187238 | G/C | 3606 | 0.228^A^ | Pro-inflammatory cytokine. Feed-back activates IFN-γ. Also known to enhance the production of IL-17, TNF-α and IL-1β. | C allele with less (stimulated) IL18 expression^G^ [12]. SNP in perfect LD (rs360717, r^2^=1) associated with altered IL-18 expression^C, F^ [13]. |
| *IL18* | rs1946518 | G/T | 3606 | 0.392 | See above. | TT reduces IL-18 level^G^ [14,15] and expression [12]. |
| *IL18* | rs360719 | A/G | 3606 | 0.228^A^ | See above. | G allele increases transcription^C, D^ [16]. Associated with development of SLE in Europeans [17]. |
| *JAK2* | rs12343867 | T/C | 3717 | 0.290 | Kinase that interact with several membrane receptors including the IFN-γ, IL-12 and IL-23 receptor | C allele associated with reduced expression^C, D, F^ [18]. |
| *NLRP1* | rs2670660 | A/G | 22861 | 0.337^A^ | Part of the NLRP1 inflammasome complex. Activation leads to processing and release of IL-1β and IL-18. Stimulates apoptosis through activation of caspase-3. | G allele reduces transcription^C, F^ [19]. |
| *NLRP1* | rs878329 | G/C | 22861 | 0.450 |  | CC genotype (vs. GG) lower mRNA levels^C, D, F^ [20]. |
| *NLRP3* | rs10754558 | C/G | 114548 | 0.358 | Part of the NLRP3 inflammasome complex. Activation leads to processing and release of IL-1β and IL-18. | G allele increased expression and mRNA stability^C, D, F^ [21]. |
| *TBX21* | rs17250932 | T/C | 30009 | 0.138^A^ | Transcription factor that controls the expression of IFN-γ. | C allele reduces *TBX21* transcription, reduces T-bet (*TBX21*) and IFN-γ level and increases IL-4 level^D, E, F^ [22]. |
| *TIRAP* | rs8177374 | C/T | 114609 | 0.170 | Adapter molecule in toll-like receptor signalling. | Increases TNF-α, IL-6, IFN-γ level^G^ [23]. |
| *TLR1* | rs4833095 | T/C | 7096 | 0.292 | Innate immune system receptor. Activates NFκB pathway. | C allele increases TLR1 expression and cell surface expression^C, E, G^ [24]. |
| *TLR5* | rs2072493 | A/G | 7100 | 0.150^B^ | Innate immune system receptor. Activates NFκB pathway. | Associated with anti-TNF outcome in a Dutch population [25]. |
| *TLR5* | rs5744174 | T/C | 7100 | 0.442 | See above. | C allele associated with higher PBMCs IFN-γ secretion to measles virus stimulaton [26], reduced CCL20 production^G^ [27], and CC reduces expression of interleukin (IL)-6 and IL-1β mRNA^F^ [28]. |

MAF: minor allele frequency - HapMap-CEU if not stated other.

^A^ 1000 Genomes Project.

^B^ Genotyping failed.

^C^ Function examined by reverse transcriptase PCR (RT-PCR)

^D^ Function examined by electrophoretic mobility shift assay (EMSA)

^E^ Function examined by flow cytometry

^F^ Function examined by luciferase reporter assay

^G^ Function examined by enzyme-linked immunosorbent assay (ELISA)

Reference List

1. Paramel GV, Folkersen L, Strawbridge RJ, Elmabsout AA, Sarndahl E, Lundman P, et al. CARD8 gene encoding a protein of innate immunity is expressed in human atherosclerosis and associated with markers of inflammation. Clin Sci (Lond). 2013 Oct;125: 401-407. CS20120572 [pii];10.1042/CS20120572 [doi].

2. Kastbom A, Johansson M, Verma D, Soderkvist P, Rantapaa-Dahlqvist S. CARD8 p.C10X polymorphism is associated with inflammatory activity in early rheumatoid arthritis. Ann Rheum Dis. 2010 Apr;69: 723-726. ard.2008.106989 [pii];10.1136/ard.2008.106989 [doi].

3. Canedo P, Corso G, Pereira F, Lunet N, Suriano G, Figueiredo C, et al. The interferon gamma receptor 1 (IFNGR1) -56C/T gene polymorphism is associated with increased risk of early gastric carcinoma. Gut. 2008 Nov;57: 1504-1508. gut.2007.143578 [pii];10.1136/gut.2007.143578 [doi].

4. Hijikata M, Shojima J, Matsushita I, Tokunaga K, Ohashi J, Hang NT, et al. Association of IFNGR2 gene polymorphisms with pulmonary tuberculosis among the Vietnamese. Hum Genet. 2012 May;131: 675-682. 10.1007/s00439-011-1112-8 [doi].

5. Wu JF, Wu TC, Chen CH, Ni YH, Chen HL, Hsu HY, et al. Serum levels of interleukin-10 and interleukin-12 predict early, spontaneous hepatitis B virus e antigen seroconversion. Gastroenterology. 2010 Jan;138: 165-172. S0016-5085(09)01659-X [pii];10.1053/j.gastro.2009.09.018 [doi].

6. Tao YP, Wang WL, Li SY, Zhang J, Shi QZ, Zhao F, et al. Associations between polymorphisms in IL-12A, IL-12B, IL-12Rbeta1, IL-27 gene and serum levels of IL-12p40, IL-27p28 with esophageal cancer. J Cancer Res Clin Oncol. 2012 Nov;138: 1891-1900. 10.1007/s00432-012-1269-0 [doi].

7. Zhao B, Meng LQ, Huang HN, Pan Y, Xu QQ. A novel functional polymorphism, 16974 A/C, in the interleukin-12-3' untranslated region is associated with risk of glioma. DNA Cell Biol. 2009 Jul;28: 335-341. 10.1089/dna.2008.0845 [doi].

8. Eskandari-Nasab E, Moghadampour M, Asadi-Saghandi A, Kharazi-nejad E, Rezaeifar A, Pourmasoumi H. Levels of Interleukin-(IL)-12p40 are Markedly Increased in Brucellosis Among Patients with Specific IL-12B Genotypes. Scand J Immunol. 2013 Jul 1;78: 85-91.

9. Cargill M, Schrodi SJ, Chang M, Garcia VE, Brandon R, Callis KP, et al. A large-scale genetic association study confirms IL12B and leads to the identification of IL23R as psoriasis-risk genes. Am J Hum Genet. 2007 Feb;80: 273-290. S0002-9297(07)62685-8 [pii];10.1086/511051 [doi].

10. Verma VK, Taneja V, Jaiswal A, Sharma S, Behera D, Sreenivas V, et al. Prevalence, distribution and functional significance of the -237C to T polymorphism in the IL-12Rbeta2 promoter in Indian tuberculosis patients. PLoS One. 2012;7: e34355. 10.1371/journal.pone.0034355 [doi];PONE-D-11-15587 [pii].

11. Kim SW, Kim ES, Moon CM, Park JJ, Kim TI, Kim WH, et al. Genetic polymorphisms of IL-23R and IL-17A and novel insights into their associations with inflammatory bowel disease. Gut. 2011 Nov;60: 1527-1536. gut.2011.238477 [pii];10.1136/gut.2011.238477 [doi].

12. Dziedziejko V, Kurzawski MF, Paczkowska E FAU - Machalinski B, Machalinski BF, Pawlik A. The impact of IL18 gene polymorphisms on mRNA levels and interleukin-18 release by peripheral blood mononuclear cells. Postepy Hig Med Dosw (Online). 2012;409-414.

13. Barbaux S, Poirier O, Godefroy T, Kleinert H, Blankenberg S, Cambien F, et al. Differential haplotypic expression of the interleukin-18 gene. Eur J Hum Genet. 2007 May 9;15: 856-863.

14. Jaiswal PK, Singh V, Srivastava P, Mittal RD. Association of IL-12, IL-18 variants and serum IL-18 with bladder cancer susceptibility in North Indian population. Gene. 2013 Apr 25;519: 128-134. S0378-1119(13)00066-8 [pii];10.1016/j.gene.2013.01.025 [doi].

15. Chen DY, Chen YM, Chen HH, Hsieh CW, Lin CC, Lan JL. Functional association of interleukin 18 gene -607 (C/A) promoter polymorphisms with disease course in Chinese patients with adult-onset Still's disease. J Rheumatol. 2009 Oct;36: 2284-2289. jrheum.090316 [pii];10.3899/jrheum.090316 [doi].

16. Sanchez E, Palomino-Morales RJ, Ortego-Centeno N, Jimenez-Alonso J, Gonzalez-Gay MA, Lopez-Nevot MA, et al. Identification of a new putative functional IL18 gene variant through an association study in systemic lupus erythematosus. Hum Mol Genet. 2009 Oct 1;18: 3739-3748. ddp301 [pii];10.1093/hmg/ddp301 [doi].

17. Song GG, Choi SJ, Ji JD, Lee YH. Association between interleukin-18 polymorphisms and systemic lupus erythematosus: a meta-analysis. Mol Biol Rep. 2012 Dec 14;40: 2581-2587. 10.1007/s11033-012-2344-y [doi].

18. Spasovski V, Tosic N, Nikcevic G, Stojiljkovic M, Zukic B, Radmilovic M, et al. The influence of novel transcriptional regulatory element in intron 14 on the expression of Janus kinase 2 gene in myeloproliferative neoplasms. J Appl Genet. 2013 Feb;54: 21-26. 10.1007/s13353-012-0125-x [doi].

19. Glinskii AB, Ma J, Ma S, Grant D, Lim CU, Sell S, et al. Identification of intergenic trans-regulatory RNAs containing a disease-linked SNP sequence and targeting cell cycle progression/differentiation pathways in multiple common human disorders. Cell Cycle. 2009 Dec;8: 3925-3942. 10113 [pii].

20. Sui J, Li H, Fang Y, Liu Y, Li M, Zhong B, et al. NLRP1 gene polymorphism influences gene transcription and is a risk factor for rheumatoid arthritis in han chinese. Arthritis Rheum. 2012 Mar;64: 647-654. 10.1002/art.33370 [doi].

21. Hitomi Y, Ebisawa M, Tomikawa M, Imai T, Komata T, Hirota T, et al. Associations of functional NLRP3 polymorphisms with susceptibility to food-induced anaphylaxis and aspirin-induced asthma. J Allergy Clin Immunol. 2009 Oct;124: 779-785.

22. Li J, Li J, You Y, Chen S. The role of upstream stimulatory factor 1 in the transcriptional regulation of the human TBX21 promoter mediated by the T-1514C polymorphism associated with systemic lupus erythematosus. Immunogenetics. 2012 May;64: 361-370. 10.1007/s00251-011-0597-6 [doi].

23. Ferwerda B, Alonso S, Banahan K, McCall MB, Giamarellos-Bourboulis EJ, Ramakers BP, et al. Functional and genetic evidence that the Mal/TIRAP allele variant 180L has been selected by providing protection against septic shock. Proc Natl Acad Sci U S A. 2009 Jun 23;106: 10272-10277. 0811273106 [pii];10.1073/pnas.0811273106 [doi].

24. Uciechowski P, Imhoff H, Lange C, Meyer CG, Browne EN, Kirsten DK, et al. Susceptibility to tuberculosis is associated with TLR1 polymorphisms resulting in a lack of TLR1 cell surface expression. J Leukoc Biol. 2011 Aug;90: 377-388. jlb.0409233 [pii];10.1189/jlb.0409233 [doi].

25. Coenen MJ, Enevold C, Barrera P, Schijvenaars MM, Toonen EJ, Scheffer H, et al. Genetic variants in toll-like receptors are not associated with rheumatoid arthritis susceptibility or anti-tumour necrosis factor treatment outcome. PLoS One. 2010;5: e14326. 10.1371/journal.pone.0014326 [doi].

26. Dhiman N, Ovsyannikova IG, Vierkant RA, Ryan JE, Shane Pankratz V, Jacobson RM, et al. Associations between SNPs in toll-like receptors and related intracellular signaling molecules and immune responses to measles vaccine: Preliminary results. Vaccine. 2008 Mar 25;26: 1731-1736.

27. Sheridan J, Mack DR, Amre DK, Israel DM, Cherkasov A, Li H, et al. A Non-Synonymous Coding Variant (L616F) in the TLR5 Gene Is Potentially Associated with Crohn's Disease and Influences Responses to Bacterial Flagellin. PLoS ONE. 2013 Apr 11;8: e61326. doi:10.1371/journal.pone.0061326.

28. Klimosch SN, Forsti A, Eckert J, Knezevic J, Bevier M, von Schonfels W, et al. Functional TLR5 Genetic Variants Affect Human Colorectal Cancer Survival. Cancer Research. 2013 Dec 15;73: 7232-7242.
