# Supplementary material for: Genetic Variations in Pattern Recognition Receptor Loci Are Associated with Anti-TNF Response in Patients with Rheumatoid Arthritis
Source: PLoS One. 2015 Oct 6;10(10):e0139781. doi: 10.1371/journal.pone.0139781 (PMC4595012; doi:10.1371/journal.pone.0139781)
Supplement: S2 Table — Adjusted odds ratio (OR)/coefficient for associations between genotypes and ACR50 and relDAS28 response to anti-TNF treatment. (a. All RA patients, b. Seropositive RA patients, c. Seronegative RA patient for TLR5 rs5744174). (DOCX) [file pone.0139781.s003.docx]

**S2a Table. ACR50 and relDAS28 anti-TNF treatment response - all RA patients.** Adjusted odds ratio (OR)/coefficient for associations between genotypes and ACR50 and relDAS28 response to anti-TNF treatment.

|  |  |  |  |  |  |
| --- | --- | --- | --- | --- | --- |
|  |  |  | ACR50 |  | relDAS28 |
|  |  |  |  | Adjusted | Adjusted |
| *Gene*  SNP | Geno-type | Freq. | Yes/no | OR (95% CI), p-, q-value | Regr. coeff. (95% CI), p-, q-value |
| *CARD8* | AA | 223 | 65/158 |  |  |
| rs2043211 | AT | 222 | 77/145 | 1.28 (0.85-1.93), 0.24, 0.75 | 0.04 (-0.02-0.09), 0.21, 0.75 |
|  | TT | 62 | 21/41 | 1.24 (0.67-2.29), 0.49, 0.76 | -0.06 (-0.14-0.03), 0.17, 0.75 |
|  | AT/TT | 284 | 98/186 | 1.27 (0.86-1.87), 0.22, 0.75 | 0.02 (-0.04-0.07), 0.58, 0.76 |
| *IFNGR1* | TT | 206 | 63/143 |  |  |
| rs2234711 | TC | 242 | 79/163 | 1.10 (0.73-1.65), 0.66, 0.75 | 0.02 (-0.04-0.08), 0.47, 0.76 |
|  | CC | 67 | 23/44 | 1.28 (0.70-2.33), 0.42, 0.87 | 0.00 (-0.08-0.08), 0.98, 0.86 |
|  | TC/CC | 309 | 102/207 | 1.13 (0.77-1.67), 0.53, 0.75 | 0.02 (-0.04-0.07), 0.56, 0.76 |
| *IFNGR2* | CC | 136 | 47/89 |  |  |
| rs17882748 | CT | 239 | 71/168 | 0.82 (0.51-1.29), 0.39, 0.86 | -0.05 (-0.11-0.02), 0.14, 0.75 |
|  | TT | 120 | 41/79 | 0.91 (0.54-1.54), 0.72, 0.76 | -0.06 (-0.13-0.02), 0.13, 0.74 |
|  | CT/TT | 359 | 112/247 | 0.85 (0.55-1.30), 0.45, 0.79 | -0.05 (-0.11-0.01), 0.095, 0.75 |
| *IFNGR2* | TT | 401 | 125/276 |  |  |
| rs8126756 | TC | 90 | 35/55 | 1.59 (0.97-2.60), 0.065, 0.75 | **0.08 (0.01-0.15), 0.027*, 0.74** |
|  | CC | 10 | 2/8 | 0.47 (0.10-2.33), 0.36, 0.92 | -0.10 (-0.29-0.09), 0.28, 0.99 |
|  | TC/CC | 100 | 37/63 | 1.42 (0.88-2.28), 0.15, 0.75 | 0.06 (-0.01-0.13), 0.077, 0.74 |
| *IL12B* | GG | 326 | 98/228 |  |  |
| rs3212217 | GC | 157 | 53/104 | 1.19 (0.79-1.82), 0.41, 0.75 | -0.02 (-0.08-0.04), 0.55, 0.76 |
|  | CC | 27 | 10/17 | 1.55 (0.66-3.60), 0.31, 0.88 | 0.01 (-0.10-0.13), 0.81, 0.90 |
|  | GC/CC | 184 | 63/121 | 1.24 (0.83-1.84), 0.29, 0.75 | -0.01 (-0.07-0.04), 0.64, 0.76 |
| *IL12B* | AA | 328 | 100/228 |  |  |
| rs3212227 | AC | 153 | 55/98 | 1.27 (0.84-1.92), 0.26, 0.75 | -0.01 (-0.07-0.05), 0.75, 0.76 |
|  | CC | 26 | 9/17 | 1.34 (0.56-3.20), 0.51, 0.90 | 0.01 (-0.11-0.13), 0.84, 0.90 |
|  | AC/CC | 179 | 64/115 | 1.28 (0.86-1.90), 0.23, 0.75 | -0.01 (-0.06-0.05), 0.83, 0.76 |
| *IL12B* | GG | 241 | 73/168 |  |  |
| rs6887695 | GC | 224 | 74/150 | 1.10 (0.74-1.64), 0.64, 0.79 | -0.05 (-0.11-0.00), 0.059, 0.74 |
|  | CC | 51 | 19/32 | 1.22 (0.63-2.34), 0.55, 0.92 | 0.03 (-0.06-0.12), 0.54, 0.90 |
|  | GC/CC | 275 | 93/182 | 1.12 (0.77-1.64), 0.56, 0.83 | -0.04 (-0.09-0.01), 0.16, 0.74 |
| *IL12RB1* | CC | 239 | 76/163 |  |  |
| rs401502 | CG | 219 | 74/145 | 1.02 (0.69-1.53), 0.91, 0.87 | 0.02 (-0.03-0.08), 0.43, 0.75 |
|  | GG | 47 | 12/35 | 0.73 (0.35-1.51), 0.40, 0.75 | 0.01 (-0.09-0.10), 0.89, 0.79 |
|  | CG/GG | 266 | 86/180 | 0.97 (0.66-1.42), 0.87, 0.76 | 0.02 (-0.03-0.07), 0.46, 0.75 |
| *IL12RB2* | CC | 516 | 165/351 |  |  |
| rs11810249 | CT | 0 | 0/0 | Not enough variants for analyses | Not enough variants for analyses |
|  | TT | 0 | 0/0 |  |  |
|  | CT/TT | 0 | 0/0 |  |  |
| *IL18* | GG | 254 | 79/175 |  |  |
| rs187238 | GC | 198 | 65/133 | 1.18 (0.78-1.77), 0.44, 0.75 | 0.03 (-0.02-0.09), 0.26, 0.75 |
|  | CC | 55 | 18/37 | 1.20 (0.63-2.27), 0.58, 0.75 | 0.07 (-0.02-0.16), 0.11, 0.76 |
|  | GC/CC | 253 | 83/170 | 1.18 (0.80-1.74), 0.40, 0.75 | 0.04 (-0.01-0.09), 0.13, 0.75 |
| *IL18* | GG | 187 | 56/131 |  |  |
| rs1946518 | GT | 246 | 86/160 | 1.32 (0.87-2.01), 0.19, 0.74 | 0.02 (-0.03-0.08), 0.44, 0.79 |
|  | TT | 82 | 24/58 | 1.08 (0.60-1.94), 0.80, 0.76 | 0.06 (-0.02-0.14), 0.15, 0.76 |
|  | GT/TT | 328 | 110/218 | 1.26 (0.85-1.88), 0.25, 0.74 | 0.03 (-0.02-0.09), 0.26, 0.76 |
| *IL18* | AA | 257 | 80/177 |  |  |
| rs360719 | AG | 199 | 65/134 | 1.18 (0.78-1.77), 0.44, 0.75 | 0.03 (-0.03-0.08), 0.36, 0.76 |
|  | GG | 55 | 18/37 | 1.20 (0.63-2.28), 0.57, 0.75 | 0.07 (-0.02-0.15), 0.14, 0.76 |
|  | AG/GG | 254 | 83/171 | 1.18 (0.80-1.73), 0.40, 0.75 | 0.03 (-0.02-0.09), 0.19, 0.75 |
| *JAK2* | TT | 244 | 81/163 |  |  |
| rs12343867 | TC | 230 | 76/154 | 1.02 (0.69-1.51), 0.91, 0.76 | 0.03 (-0.03-0.08), 0.35, 0.76 |
|  | CC | 41 | 9/32 | 0.57 (0.25-1.26), 0.16, 0.75 | 0.03 (-0.07-0.13), 0.50, 0.76 |
|  | TC/CC | 271 | 85/186 | 0.94 (0.64-1.38), 0.75, 0.87 | 0.03 (-0.03-0.08), 0.31, 0.75 |
| *NLRP1* | AA | 151 | 42/109 |  |  |
| rs2670660 | AG | 250 | 86/164 | 1.37 (0.87-2.15), 0.17, 0.83 | 0.00 (-0.06-0.06), 0.92, 0.86 |
|  | GG | 109 | 35/74 | 1.09 (0.63-1.89), 0.76, 0.90 | -0.01 (-0.09-0.06), 0.70, 0.90 |
|  | AG/GG | 359 | 121/238 | 1.28 (0.83-1.96), 0.26, 0.88 | 0.00 (-0.06-0.06), 0.94, 0.86 |
| *NLRP1* | GG | 149 | 47/102 |  |  |
| rs878329 | GC | 261 | 86/175 | 1.06 (0.68-1.65), 0.80, 0.87 | 0.00 (-0.06-0.06), 0.89, 0.76 |
|  | CC | 102 | 30/72 | 0.80 (0.45-1.40), 0.43, 0.76 | -0.01 (-0.08-0.07), 0.83, 0.99 |
|  | GC/CC | 363 | 116/247 | 0.98 (0.64-1.49), 0.91, 0.79 | -0.01 (-0.06-0.05), 0.86, 0.76 |
| *NLRP3* | CC | 181 | 60/121 |  |  |
| rs10754558 | CG | 243 | 79/164 | 0.88 (0.58-1.35), 0.57, 0.88 | **-0.07 (-0.13-(-)0.01), 0.023*, 0.74** |
|  | GG | 85 | 23/62 | 0.73 (0.41-1.32), 0.30, 0.75 | -0.04 (-0.12-0.03), 0.28, 0.76 |
|  | CG/GG | 328 | 102/226 | 0.84 (0.57-1.26), 0.41, 0.76 | **-0.06 (-0.12--0.01), 0.029*, 0.74** |
| *TBX21* | TT | 344 | 116/228 |  |  |
| rs17250932 | TC | 151 | 44/107 | 0.75 (0.49-1.14), 0.18, 0.76 | -0.01 (-0.07-0.04), 0.62, 0.99 |
|  | CC | 8 | 3/5 | 1.44 (0.32-6.50), 0.63, 0.75 | 0.15 (-0.06-0.37), 0.16, 0.75 |
|  | TC/CC | 159 | 47/112 | 0.77 (0.51-1.17), 0.22, 0.79 | -0.01 (-0.06-0.05), 0.83, 0.88 |
| *TIRAP* | CC | 409 | 129/280 |  |  |
| rs8177374 | CT | 99 | 37/62 | 1.22 (0.77-1.96), 0.40, 0.93 | 0.02 (-0.05-0.09), 0.53, 0.76 |
|  | TT | 5 | 0/5 | - | -0.08 (-0.34-0.19), 0.56, 0.76 |
|  | CT/TT | 104 | 37/67 | 1.14 (0.71-1.81), 0.59, 0.86 | 0.02 (-0.05-0.08), 0.62, 0.76 |
| ***TLR1*** | TT | 312 | 90/222 |  |  |
| **rs4833095** | TC | 178 | 63/115 | 1.42 (0.95-2.13), 0.09, 0.76 | 0.00 (-0.05-0.06), 0.90, 0.79 |
|  | CC | 21 | 12/9 | **2.93 (1.17-7.33), 0.022*, 0.75** | 0.07 (-0.07-0.20), 0.33, 0.79 |
|  | TC/CC | 199 | 75/124 | **1.54 (1.05-2.28), 0.029*, 0.75** | 0.01 (-0.04-0.06), 0.71, 0.86 |
| *TLR5* | TT | 170 | 49/121 |  |  |
| rs5744174 | TC | 234 | 78/156 | 1.35 (0.87-2.11), 0.18, 0.75 | 0.04 (-0.02-0.10), 0.19, 0.76 |
|  | CC | 107 | 38/69 | 1.51 (0.88-2.57), 0.13, 0.76 | **0.08 (0.01-0.15), 0.033*, 0.75** |
|  | TC/CC | 341 | 116/225 | 1.40 (0.93-2.12), 0.11, 0.75 | 0.05 (0.00-0.11), 0.067, 0.75 |

Logistic regression, adjusted for gender, HAQ-, DAS28-, DMARD at baseline. OR: odds ratio; EULAR, G/M/N: European League Against Rheumatism response criteria, good/moderate/none. Freq.: frequency. Correction for multiple testing using False Discovery Rate classical one-stage method set at 0.05 (q-value), based on 113 tests in analyses of secondary outcomes.

**S2b Table. ACR50 and relDAS28 anti-TNF treatment response - seropositive RA patients.** Adjusted odds ratio (OR)/coefficient for associations between genotypes and ACR50 and relDAS28 response to anti-TNF treatment.

|  |  |  |  |  |  |
| --- | --- | --- | --- | --- | --- |
|  |  |  | ACR50 |  | relDAS28 |
|  |  |  |  | Adjusted | Adjusted |
| *Gene*  SNP | Geno-type | Freq. | Yes/no | OR (95% CI), p-,q-value | Regr. coeff. (95% CI), p-, q-value |
| *CARD8* | AA | 170 | 49/121 |  |  |
| rs2043211 | AT | 165 | 60/105 | 1.38 (0.86-2.22), 0.19, 0.75 | 0.05 (-0.02-0.12), 0.15, 0.75 |
|  | TT | 47 | 17/30 | 1.40 (0.69-2.83), 0.35, 0.76 | -0.06 (-0.17-0.04), 0.22, 0.75 |
|  | AT/TT | 212 | 77/135 | 1.38 (0.88-2.17), 0.16, 0.75 | 0.02 (-0.04-0.09), 0.46, 0.76 |
| *IFNGR1* | TT | 158 | 47/111 |  |  |
| rs2234711 | TC | 187 | 67/120 | 1.30 (0.82-2.08), 0.27, 0.75 | 0.02 (-0.04-0.09), 0.46, 0.76 |
|  | CC | 45 | 14/31 | 1.13 (0.54-2.37), 0.75, 0.87 | 0.02 (-0.08-0.13), 0.70, 0.86 |
|  | TC/CC | 232 | 81/151 | 1.27 (0.81-1.99), 0.30, 0.75 | 0.02 (-0.04-0.09), 0.46, 0.76 |
| *IFNGR2* | CC | 105 | 36/69 |  |  |
| rs17882748 | CT | 184 | 59/125 | 0.90 (0.53-1.52), 0.69, 0.86 | -0.04 (-0.12-0.03), 0.29, 0.75 |
|  | TT | 87 | 28/59 | 0.79 (0.42-1.47), 0.45, 0.76 | **-0.10 (-0.19-(-)0.01), 0.026*, 0.74** |
|  | CT/TT | 271 | 87/184 | 0.86 (0.52-1.41), 0.55, 0.79 | -0.06 (-0.13-0.01), 0.097, 0.75 |
| *IFNGR2* | TT | 304 | 97/207 |  |  |
| rs8126756 | TC | 68 | 27/41 | 1.61 (0.91-2.84), 0.10, 0.75 | **0.09 (0.01-0.18), 0.029*, 0.74** |
|  | CC | 7 | 2/5 | 0.88 (0.16-4.87), 0.88, 0.92 | 0.00 (-0.23-0.24), 0.98, 0.99 |
|  | TC/CC | 75 | 29/46 | 1.52 (0.88-2.64), 0.13, 0.75 | **0.08 (0.00-0.16), 0.039*, 0.74** |
| *IL12B* | GG | 244 | 74/170 |  |  |
| rs3212217 | GC | 122 | 45/77 | 1.30 (0.80-2.09), 0.29, 0.75 | -0.03 (-0.10-0.04), 0.43, 0.76 |
|  | CC | 20 | 6/14 | 1.15 (0.41-3.27), 0.79, 0.88 | -0.02 (-0.16-0.13), 0.82, 0.90 |
|  | GC/CC | 142 | 51/91 | 1.28 (0.81-2.01), 0.29, 0.75 | -0.03 (-0.09-0.04), 0.44, 0.76 |
| *IL12B* | AA | 245 | 76/169 |  |  |
| rs3212227 | AC | 119 | 46/73 | 1.32 (0.82-2.13), 0.25, 0.75 | -0.02 (-0.09-0.05), 0.51, 0.76 |
|  | CC | 20 | 6/14 | 1.11 (0.39-3.13), 0.85, 0.90 | -0.02 (-0.16-0.13), 0.82, 0.90 |
|  | AC/CC | 139 | 52/87 | 1.29 (0.82-2.04), 0.27, 0.75 | -0.02 (-0.09-0.04), 0.51, 0.76 |
| *IL12B* | GG | 178 | 55/123 |  |  |
| rs6887695 | GC | 177 | 63/114 | 1.15 (0.73-1.82), 0.55, 0.79 | **-0.07 (-0.13-0.00), 0.035*, 0.74** |
|  | CC | 35 | 11/24 | 0.94 (0.42-2.11), 0.89, 0.92 | -0.01 (-0.13-0.10), 0.83, 0.90 |
|  | GC/CC | 212 | 74/138 | 1.11 (0.72-1.73), 0.63, 0.83 | -0.06 (-0.12-0.00), 0.057, 0.74 |
| *IL12RB1* | CC | 180 | 62/118 |  |  |
| rs401502 | CG | 166 | 57/109 | 0.92 (0.58-1.46), 0.73, 0.87 | 0.03 (-0.03-0.10), 0.31, 0.75 |
|  | GG | 35 | 7/28 | 0.54 (0.22-1.34), 0.19, 0.75 | 0.03 (-0.08-0.15), 0.59, 0.79 |
|  | CG/GG | 201 | 64/137 | 0.85 (0.55-1.33), 0.48, 0.76 | 0.03 (-0.03-0.10), 0.29, 0.75 |
| *IL12RB2* | CC | 390 | 128/262 |  |  |
| rs11810249 | CT | 0 | 0/0 | Not enough variants for analyses | Not enough variants for analyses |
|  | TT | 0 | 0/0 |  |  |
|  | CT/TT | 0 | 0/0 |  |  |
| *IL18* | GG | 188 | 56/132 |  |  |
| rs187238 | GC | 154 | 53/101 | 1.40 (0.87-2.26), 0.16, 0.75 | 0.04 (-0.03-0.10), 0.27, 0.75 |
|  | CC | 40 | 16/24 | 1.83 (0.87-3.83), 0.11, 0.75 | 0.05 (-0.05-0.16), 0.33, 0.76 |
|  | GC/CC | 194 | 69/125 | 1.48 (0.95-2.33), 0.086, 0.75 | 0.04 (-0.02-0.10), 0.21, 0.75 |
| *IL18* | GG | 138 | 39/99 |  |  |
| rs1946518 | GT | 190 | 70/120 | **1.64 (1-2.68), 0.048*, 0.74** | 0.02 (-0.05-0.09), 0.59, 0.79 |
|  | TT | 63 | 20/43 | 1.36 (0.69-2.68), 0.37, 0.76 | 0.04 (-0.05-0.14), 0.39, 0.76 |
|  | GT/TT | 253 | 90/163 | 1.57 (0.98-2.51), 0.059, 0.74 | 0.02 (-0.04-0.09), 0.46, 0.76 |
| *IL18* | AA | 191 | 57/134 |  |  |
| rs360719 | AG | 156 | 54/102 | 1.43 (0.89-2.29), 0.14, 0.75 | 0.03 (-0.03-0.10), 0.32, 0.76 |
|  | GG | 40 | 16/24 | 1.84 (0.88-3.85), 0.11, 0.75 | 0.05 (-0.06-0.16), 0.39, 0.76 |
|  | AG/GG | 196 | 70/126 | 1.50 (0.96-2.35), 0.075, 0.75 | 0.04 (-0.03-0.10), 0.26, 0.75 |
| *JAK2* | TT | 180 | 59/121 |  |  |
| rs12343867 | TC | 174 | 62/112 | 1.19 (0.76-1.89), 0.45, 0.76 | 0.03 (-0.03-0.10), 0.36, 0.76 |
|  | CC | 36 | 8/28 | 0.63 (0.26-1.49), 0.29, 0.75 | 0.05 (-0.06-0.17), 0.36, 0.76 |
|  | TC/CC | 210 | 70/140 | 1.08 (0.70-1.68), 0.73, 0.87 | 0.03 (-0.03-0.10), 0.28, 0.75 |
| *NLRP1* | AA | 113 | 35/78 |  |  |
| rs2670660 | AG | 195 | 66/129 | 1.13 (0.68-1.89), 0.64, 0.83 | -0.01 (-0.09-0.06), 0.70, 0.86 |
|  | GG | 77 | 26/51 | 0.93 (0.49-1.77), 0.83, 0.90 | -0.01 (-0.10-0.08), 0.84, 0.90 |
|  | AG/GG | 272 | 92/180 | 1.07 (0.66-1.74), 0.79, 0.88 | -0.01 (-0.08-0.06), 0.71, 0.86 |
| *NLRP1* | GG | 110 | 37/73 |  |  |
| rs878329 | GC | 206 | 67/139 | 0.92 (0.55-1.53), 0.74, 0.87 | -0.03 (-0.10-0.04), 0.42, 0.76 |
|  | CC | 70 | 22/48 | 0.74 (0.38-1.44), 0.37, 0.76 | 0.00 (-0.09-0.09), 0.99, 0.99 |
|  | GC/CC | 276 | 89/187 | 0.87 (0.53-1.41), 0.57, 0.79 | -0.02 (-0.09-0.05), 0.52, 0.76 |
| *NLRP3* | CC | 138 | 46/92 |  |  |
| rs10754558 | CG | 184 | 64/120 | 0.93 (0.57-1.52), 0.77, 0.88 | **-0.08 (-0.14-(-)0.01), 0.034*, 0.74** |
|  | GG | 63 | 16/47 | 0.66 (0.33-1.32), 0.24, 0.75 | -0.04 (-0.13-0.06), 0.42, 0.76 |
|  | CG/GG | 247 | 80/167 | 0.86 (0.54-1.36), 0.52, 0.76 | **-0.07 (-0.13-0.00), 0.049*, 0.74** |
| *TBX21* | TT | 264 | 87/177 |  |  |
| rs17250932 | TC | 114 | 36/78 | 0.83 (0.51-1.35), 0.45, 0.76 | 0.00 (-0.07-0.07), 0.99, 0.99 |
|  | CC | 6 | 3/3 | 2.52 (0.44-14.36), 0.3, 0.75 | 0.20 (-0.06-0.45), 0.13, 0.75 |
|  | TC/CC | 120 | 39/81 | 0.88 (0.54-1.41), 0.58, 0.79 | 0.01 (-0.06-0.08), 0.77, 0.88 |
| *TIRAP* | CC | 312 | 105/207 |  |  |
| rs8177374 | CT | 74 | 25/49 | 0.97 (0.55-1.69), 0.91, 0.93 | 0.03 (-0.05-0.11), 0.40, 0.76 |
|  | TT | 4 | 0/4 | - | -0.12 (-0.43-0.18), 0.43, 0.76 |
|  | CT/TT | 78 | 25/53 | 0.89 (0.51-1.54), 0.68, 0.86 | 0.03 (-0.05-0.10), 0.51, 0.76 |
| ***TLR1*** | TT | 235 | 73/162 |  |  |
| **rs4833095** | TC | 136 | 46/90 | 1.21 (0.76-1.93), 0.43, 0.76 | -0.02 (-0.09-0.05), 0.56, 0.79 |
|  | CC | 15 | 9/6 | 2.48 (0.83-7.43), 0.10, 0.75 | 0.05 (-0.12-0.21), 0.56, 0.79 |
|  | TC/CC | 151 | 55/96 | 1.31 (0.84-2.06), 0.24, 0.75 | -0.01 (-0.08-0.05), 0.70, 0.86 |
| *TLR5* | TT | 126 | 40/86 |  |  |
| rs5744174 | TC | 176 | 60/116 | 1.32 (0.79-2.20), 0.29, 0.75 | 0.02 (-0.05-0.10), 0.50, 0.76 |
|  | CC | 86 | 29/57 | 1.25 (0.68-2.29), 0.48, 0.76 | 0.06 (-0.03-0.15), 0.17, 0.75 |
|  | TC/CC | 262 | 89/173 | 1.29 (0.80-2.08), 0.29, 0.75 | 0.04 (-0.03-0.10), 0.29, 0.75 |

Logistic regression, adjusted for gender, HAQ-, DAS28-, DMARD at baseline. OR: odds ratio; EULAR, G/M/N: European League Against Rheumatism response criteria, good/moderate/none. Freq.: frequency. Correction for multiple testing using False Discovery Rate classical one-stage method set at 0.05 (q-value), based on 119 tests in IgM-rheumatoid factor stratified analyses.

**S2c Table. ACR50 and relDAS28 anti-TNF treatment response - seronegative RA patients.** Adjusted odds ratio (OR)/coefficient for associations between genotypes and ACR50 and relDAS28 response to anti-TNF treatment.

|  |  |  |  |  |  |
| --- | --- | --- | --- | --- | --- |
|  |  |  | ACR50 |  | relDAS28 |
|  |  |  |  | Adjusted | Adjusted |
| *Gene*  SNP | Geno-type | Freq. | Yes/no | OR (95% CI), p-, q-value | Regr. coeff. (95% CI), p-, q-value |
| ***TLR5*** | TT | 44 | 9/35 |  |  |
| **rs5744174** | TC | 58 | 18/40 | 1.90 (0.73-4.96), 0.19, 0.70 | 0.10 (0.00-0.20), 0.055, 0.54 |
|  | CC | 21 | 9/12 | **3.51 (1.07-11.5), 0.038*, 0.54** | **0.15 (0.01-0.28), 0.035*, 0.54** |
|  | TC/CC | 79 | 27/52 | 2.26 (0.92-5.58), 0.077, 0.61 | **0.11 (0.02-0.21), 0.022*, 0.54** |

Logistic regression, adjusted for gender, HAQ-, DAS28-, DMARD at baseline. OR: odds ratio; EULAR, G/M/N: European League Against Rheumatism response criteria, good/moderate/none. Freq.: frequency. Correction for multiple testing using False Discovery Rate classical one-stage method set at 0.05 (q-value), based on 119 tests in IgM-rheumatoid factor stratified analyses.
