# Supplementary material for: Genetic Variations in Pattern Recognition Receptor Loci Are Associated with Anti-TNF Response in Patients with Rheumatoid Arthritis
Source: PLoS One. 2015 Oct 6;10(10):e0139781. doi: 10.1371/journal.pone.0139781 (PMC4595012; doi:10.1371/journal.pone.0139781)
Supplement: S3 Table — Adjusted odds ratios for associations between genotypes and EULAR anti-TNF treatment response. (a. All RA patients, b. Seropositive RA patients, c. Seronegative RA patient for TLR5 rs5744174). (DOCX) [file pone.0139781.s004.docx]

**S3a Table. EULAR anti-TNF treatment response - all RA patients.** Adjusted odds ratios for associations between genotypes and EULAR anti-TNF treatment response.

|  |  |  |  |  |  |
| --- | --- | --- | --- | --- | --- |
|  |  |  |  | G&M vs. N | G vs. M&N |
|  |  |  |  | Adjusted | Adjusted |
| *Gene*  SNP | Genotype | Freq. | G/M/N | OR (95% CI), p-, q-value | OR (95% CI), p-, q-value |
| *CARD8* | AA | 223 | 86/70/67 |  |  |
| rs2043211 | AT | 222 | 102/62/58 | 1.23 (0.80-1.9), 0.34, 0.71 | 1.40 (0.95-2.05), 0.092, 0.47 |
|  | TT | 62 | 26/11/25 | 0.58 (0.32-1.06), 0.079, 0.47 | 1.10 (0.61-1.98), 0.75, 0.89 |
|  | AT/TT | 284 | 128/73/83 | 1.03 (0.69-1.53), 0.90, 0.92 | 1.32 (0.92-1.91), 0.13, 0.48 |
| *IFNGR1* | TT | 206 | 82/64/60 |  |  |
| rs2234711 | TC | 242 | 105/69/68 | 1.04 (0.68-1.59), 0.87, 0.92 | 1.15 (0.78-1.69), 0.48, 0.72 |
|  | CC | 67 | 31/13/23 | 0.82 (0.44-1.50), 0.51, 0.74 | 1.29 (0.73-2.28), 0.38, 0.72 |
|  | TC/CC | 309 | 136/82/91 | 0.98 (0.65-1.47), 0.92, 0.94 | 1.18 (0.82-1.70), 0.38, 0.72 |
| *IFNGR2* | CC | 136 | 61/39/36 |  |  |
| rs17882748 | CT | 239 | 103/60/76 | 0.80 (0.49-1.31), 0.38, 0.72 | 0.97 (0.63-1.50), 0.89, 0.92 |
|  | TT | 120 | 46/36/38 | 0.66 (0.37-1.16), 0.15, 0.50 | 0.74 (0.45-1.24), 0.26, 0.64 |
|  | CT/TT | 359 | 149/96/114 | 0.75 (0.48-1.19), 0.23, 0.61 | 0.89 (0.59-1.34), 0.57, 0.77 |
| *IFNGR2* | TT | 401 | 165/116/120 |  |  |
| rs8126756 | TC | 90 | 44/23/23 | 1.53 (0.88-2.67), 0.13, 0.48 | 1.43 (0.89-2.30), 0.14, 0.48 |
|  | CC | 10 | 3/2/5 | 0.34 (0.09-1.27), 0.11, 0.48 | 0.59 (0.14-2.42), 0.46, 0.72 |
|  | TC/CC | 100 | 47/25/28 | 1.28 (0.76-2.15), 0.35, 0.71 | 1.32 (0.84-2.07), 0.23, 0.61 |
| *IL12B* | GG | 326 | 138/95/93 |  |  |
| rs3212217 | GC | 157 | 64/42/51 | 0.82 (0.53-1.26), 0.37, 0.72 | 0.90 (0.61-1.34), 0.62, 0.81 |
|  | CC | 27 | 14/7/6 | 1.71 (0.64-4.61), 0.29, 0.66 | 1.67 (0.74-3.78), 0.22, 0.59 |
|  | GC/CC | 184 | 78/49/57 | 0.91 (0.60-1.37), 0.65, 0.83 | 0.99 (0.68-1.44), 0.96, 0.97 |
| *IL12B* | AA | 328 | 139/95/94 |  |  |
| rs3212227 | AC | 153 | 64/42/47 | 0.88 (0.57-1.36), 0.55, 0.75 | 0.94 (0.63-1.40), 0.75, 0.89 |
|  | CC | 26 | 14/6/6 | 1.63 (0.60-4.42), 0.34, 0.71 | 1.80 (0.78-4.14), 0.17, 0.52 |
|  | AC/CC | 179 | 78/48/53 | 0.96 (0.63-1.45), 0.83, 0.91 | 1.03 (0.71-1.50), 0.87, 0.92 |
| ***IL12B*** | GG | 241 | 109/70/62 |  |  |
| **rs6887695** | GC | 224 | 91/54/79 | **0.60 (0.40-0.91), 0.017*, 0.46** | 0.82 (0.56-1.20), 0.31, 0.70 |
|  | CC | 51 | 20/22/9 | 1.44 (0.64-3.22), 0.38, 0.72 | 0.81 (0.43-1.53), 0.52, 0.74 |
|  | GC/CC | 275 | 111/76/88 | 0.69 (0.46-1.03), 0.068, 0.47 | 0.82 (0.57-1.17), 0.28, 0.65 |
| *IL12RB1* | CC | 239 | 99/68/72 |  |  |
| rs401502 | CG | 219 | 99/61/59 | 1.09 (0.71-1.66), 0.70, 0.87 | 1.10 (0.76-1.61), 0.61, 0.81 |
|  | GG | 47 | 19/13/15 | 0.95 (0.47-1.91), 0.88, 0.92 | 0.93 (0.48-1.78), 0.82, 0.91 |
|  | CG/GG | 266 | 118/74/74 | 1.06 (0.71-1.59), 0.77, 0.91 | 1.07 (0.75-1.54), 0.71, 0.87 |
| *IL12RB2* | CC | 516 | 219/146/151 |  |  |
| rs11810249 | CT | 0 | 0/0/0 | Not enough variants for analyses | Not enough variants for analyses |
|  | TT | 0 | 0/0/0 |  |  |
|  | CT/TT | 0 | 0/0/0 |  |  |
| *IL18* | GG | 254 | 96/76/82 |  |  |
| rs187238 | GC | 198 | 91/51/56 | 1.39 (0.91-2.13), 0.13, 0.48 | **1.50 (1.02-2.21), 0.041*, 0.46** |
|  | CC | 55 | 26/16/13 | 1.85 (0.91-3.74), 0.089, 0.47 | 1.58 (0.86-2.90), 0.14, 0.48 |
|  | GC/CC | 253 | 117/67/69 | 1.48 (0.99-2.21), 0.057, 0.47 | **1.52 (1.05-2.19), 0.026*, 0.46** |
| *IL18* | GG | 187 | 73/50/64 |  |  |
| rs1946518 | GT | 246 | 111/70/65 | **1.57 (1.02-2.43), 0.041*, 0.46** | 1.36 (0.92-2.03), 0.13, 0.48 |
|  | TT | 82 | 36/24/22 | 1.72 (0.94-3.16), 0.077, 0.47 | 1.25 (0.73-2.16), 0.42, 0.72 |
|  | GT/TT | 328 | 147/94/87 | **1.61 (1.07-2.42), 0.022*, 0.46** | 1.34 (0.92-1.94), 0.13, 0.48 |
| *IL18* | AA | 257 | 99/77/81 |  |  |
| rs360719 | AG | 199 | 91/52/56 | 1.35 (0.88-2.06), 0.17, 0.53 | 1.41 (0.96-2.08), 0.079, 0.47 |
|  | GG | 55 | 26/16/13 | 1.77 (0.87-3.60), 0.11, 0.48 | 1.49 (0.81-2.75), 0.20, 0.57 |
|  | AG/GG | 254 | 117/68/69 | 1.43 (0.95-2.13), 0.084, 0.47 | 1.43 (0.99-2.06), 0.054, 0.47 |
| *JAK2* | TT | 244 | 107/57/80 |  |  |
| rs12343867 | TC | 230 | 95/74/61 | 1.45 (0.96-2.21), 0.078, 0.47 | 0.96 (0.66-1.39), 0.82, 0.91 |
|  | CC | 41 | 19/12/10 | 1.57 (0.72-3.45), 0.26, 0.64 | 1.09 (0.55-2.14), 0.81, 0.91 |
|  | TC/CC | 271 | 114/86/71 | 1.47 (0.99-2.19), 0.058, 0.47 | 0.98 (0.68-1.40), 0.90, 0.92 |
| *NLRP1* | AA | 151 | 62/41/48 |  |  |
| rs2670660 | AG | 250 | 113/63/74 | 1.10 (0.69-1.73), 0.69, 0.87 | 1.19 (0.78-1.81), 0.41, 0.72 |
|  | GG | 109 | 42/41/26 | 1.26 (0.71-2.25), 0.43, 0.72 | 0.83 (0.49-1.39), 0.48, 0.72 |
|  | AG/GG | 359 | 155/104/100 | 1.14 (0.74-1.76), 0.55, 0.75 | 1.07 (0.72-1.58), 0.75, 0.89 |
| *NLRP1* | GG | 149 | 60/41/48 |  |  |
| rs878329 | GC | 261 | 116/69/76 | 1.16 (0.74-1.84), 0.51, 0.74 | 1.17 (0.77-1.78), 0.47, 0.72 |
|  | CC | 102 | 40/36/26 | 1.19 (0.66-2.15), 0.55, 0.75 | 0.88 (0.52-1.50), 0.64, 0.83 |
|  | GC/CC | 363 | 156/105/102 | 1.17 (0.76-1.81), 0.47, 0.72 | 1.08 (0.73-1.61), 0.71, 0.87 |
| *NLRP3* | CC | 181 | 86/50/45 |  |  |
| rs10754558 | CG | 243 | 96/69/78 | **0.61 (0.39-0.96), 0.033*, 0.46** | 0.71 (0.48-1.06), 0.098, 0.48 |
|  | GG | 85 | 35/24/26 | 0.75 (0.41-1.36), 0.34, 0.71 | 0.80 (0.47-1.37), 0.42, 0.72 |
|  | CG/GG | 328 | 131/93/104 | **0.64 (0.42-0.99), 0.045*, 0.46** | 0.74 (0.50-1.07), 0.11, 0.48 |
| *TBX21* | TT | 344 | 148/90/106 |  |  |
| rs17250932 | TC | 151 | 64/48/39 | 1.16 (0.74-1.81), 0.52, 0.74 | 0.95 (0.64-1.41), 0.80, 0.91 |
|  | CC | 8 | 3/3/2 | 1.79 (0.33-9.78), 0.50, 0.74 | 1.00 (0.22-4.50), 1.00, 1.00 |
|  | TC/CC | 159 | 67/51/41 | 1.18 (0.76-1.84), 0.45, 0.72 | 0.95 (0.65-1.41), 0.81, 0.91 |
| *TIRAP* | CC | 409 | 180/105/124 |  |  |
| rs8177374 | CT | 99 | 40/36/23 | 1.35 (0.79-2.29), 0.27, 0.65 | 0.82 (0.52-1.30), 0.40, 0.72 |
|  | TT | 5 | 0/2/3 | 0.30 (0.05-1.93), 0.20, 0.58 | - |
|  | CT/TT | 104 | 40/38/26 | 1.23 (0.74-2.05), 0.43, 0.72 | 0.76 (0.49-1.20), 0.24, 0.62 |
| ***TLR1*** | TT | 312 | 130/91/91 |  |  |
| **rs4833095** | TC | 178 | 75/53/50 | 1.17 (0.76-1.80), 0.47, 0.72 | 1.06 (0.72-1.55), 0.78, 0.91 |
|  | CC | 21 | 14/2/5 | 1.09 (0.37-3.20), 0.87, 0.92 | **2.80 (1.07-7.35), 0.037*, 0.46** |
|  | TC/CC | 199 | 89/55/55 | 1.16 (0.77-1.76), 0.48, 0.72 | 1.17 (0.81-1.69), 0.41, 0.72 |
| ***TLR5*** | TT | 170 | 61/55/54 |  |  |
| **rs5744174** | TC | 234 | 104/62/68 | 1.25 (0.80-1.96), 0.33, 0.71 | **1.55 (1.02-2.35), 0.040*, 0.46** |
|  | CC | 107 | 53/26/28 | 1.51 (0.86-2.66), 0.16, 0.50 | **1.96 (1.18-3.25), 0.009**, 0.46** |
|  | TC/CC | 341 | 157/88/96 | 1.32 (0.87-2.02), 0.19, 0.57 | **1.67 (1.13-2.46), 0.010*, 0.46** |

| Logistic regression, adjusted for gender, HAQ-, DAS28-, DMARD at baseline. OR: odds ratio; EULAR, G/M/N: European League Against Rheumatism response criteria, good/moderate/none. Freq.: frequency. Correction for multiple testing using False Discovery Rate classical one-stage method set at 0.05 (q-value), based on 113 tests in analyses of primary outcome. |
| --- |

**S3b Table. EULAR anti-TNF treatment response - seropositive RA patients.** Adjusted odds ratios for associations between genotypes and EULAR anti-TNF treatment response.

|  |  |  |  |  |  |
| --- | --- | --- | --- | --- | --- |
|  |  |  |  | G&M vs. N | G vs. M&N |
|  |  |  |  | Adjusted | Adjusted |
| Gene  (SNP) | Geno-type | Freq. | G/M/N | OR (95% CI), p-, q-value | OR (95% CI), p-, q-value |
| ***CARD8*** | AA | 170 | 64/53/53 |  |  |
| **rs2043211** | AT | 165 | 81/42/42 | 1.37 (0.83-2.27), 0.22, 0.6 | **1.60 (1.02-2.49), 0.039*, 0.40** |
|  | TT | 47 | 20/8/19 | 0.59 (0.3-1.19), 0.14, 0.55 | 1.19 (0.61-2.33), 0.60, 0.99 |
|  | AT/TT | 212 | 101/50/61 | 1.11 (0.7-1.76), 0.65, 0.99 | 1.50 (0.98-2.28), 0.059, 0.48 |
| *IFNGR1* | TT | 158 | 61/50/47 |  |  |
| rs2234711 | TC | 187 | 86/47/54 | 1.00 (0.61-1.62), 0.99, 0.99 | 1.32 (0.85-2.05), 0.21, 0.58 |
|  | CC | 45 | 22/9/14 | 0.97 (0.46-2.05), 0.93, 0.99 | 1.46 (0.74-2.89), 0.28, 0.67 |
|  | TC/CC | 232 | 108/56/68 | 0.99 (0.62-1.58), 0.97, 0.99 | 1.35 (0.89-2.05), 0.16, 0.55 |
| *IFNGR2* | CC | 105 | 48/29/28 |  |  |
| rs17882748 | CT | 184 | 85/46/53 | 0.94 (0.53-1.65), 0.83, 0.99 | 1.06 (0.64-1.73), 0.83, 0.99 |
|  | TT | 87 | 30/23/34 | **0.44 (0.23-0.84), 0.013*, 0.31** | 0.57 (0.31-1.05), 0.07, 0.49 |
|  | CT/TT | 271 | 115/69/87 | 0.73 (0.43-1.24), 0.24, 0.63 | 0.87 (0.55-1.39), 0.56, 0.99 |
| *IFNGR2* | TT | 304 | 127/85/92 |  |  |
| rs8126756 | TC | 68 | 34/18/16 | 1.72 (0.89-3.32), 0.11, 0.52 | 1.48 (0.86-2.55), 0.16, 0.55 |
|  | CC | 7 | 3/0/4 | 0.30 (0.06-1.46), 0.14, 0.54 | 1.10 (0.23-5.36), 0.90, 0.99 |
|  | TC/CC | 75 | 37/18/20 | 1.40 (0.76-2.56), 0.28, 0.67 | 1.44 (0.86-2.43), 0.17, 0.55 |
| *IL12B* | GG | 244 | 105/69/70 |  |  |
| rs3212217 | GC | 122 | 52/32/38 | 0.85 (0.52-1.40), 0.53, 0.96 | 0.91 (0.58-1.43), 0.68, 0.99 |
|  | CC | 20 | 11/3/6 | 1.17 (0.40-3.38), 0.77, 0.99 | 1.90 (0.73-4.95), 0.19, 0.57 |
|  | GC/CC | 142 | 63/35/44 | 0.89 (0.56-1.44), 0.64, 0.99 | 1.01 (0.66-1.55), 0.97, 0.99 |
| *IL12B* | AA | 245 | 106/69/70 |  |  |
| rs3212227 | AC | 119 | 52/32/35 | 0.90 (0.54-1.49), 0.68, 0.99 | 0.93 (0.59-1.47), 0.77, 0.99 |
|  | CC | 20 | 11/3/6 | 1.15 (0.40-3.30), 0.80, 0.99 | 1.87 (0.72-4.87), 0.20, 0.58 |
|  | AC/CC | 139 | 63/35/41 | 0.93 (0.58-1.51), 0.78, 0.99 | 1.03 (0.67-1.59), 0.89, 0.99 |
| *IL12B* | GG | 178 | 83/50/45 |  |  |
| rs6887695 | GC | 177 | 76/41/60 | **0.61 (0.38-0.99), 0.046*, 0.42** | 0.84 (0.55-1.29), 0.43, 0.83 |
|  | CC | 35 | 12/15/8 | 1.07 (0.44-2.64), 0.88, 0.99 | 0.61 (0.28-1.32), 0.21, 0.58 |
|  | GC/CC | 212 | 88/56/68 | 0.67 (0.42-1.06), 0.087, 0.52 | 0.80 (0.53-1.21), 0.28, 0.67 |
| *IL12RB1* | CC | 180 | 78/47/55 |  |  |
| rs401502 | CG | 166 | 77/46/43 | 1.18 (0.72-1.94), 0.51, 0.94 | 1.05 (0.68-1.62), 0.84, 0.99 |
|  | GG | 35 | 14/9/12 | 0.98 (0.44-2.19), 0.96, 0.99 | 0.93 (0.44-1.97), 0.85, 0.99 |
|  | CG/GG | 201 | 91/55/55 | 1.14 (0.71-1.81), 0.59, 0.99 | 1.03 (0.68-1.55), 0.90, 0.99 |
| *IL12RB2* | CC | 390 | 170/106/114 |  |  |
| rs11810249 | CT | 0 | 0/0/0 | Not enough variants for analyses | Not enough variants for analyses |
|  | TT | 0 | 0/0/0 |  |  |
|  | CT/TT | 0 | 0/0/0 |  |  |
| ***IL18*** | GG | 188 | 70/58/60 |  |  |
| **rs187238** | GC | 154 | 74/37/43 | 1.43 (0.87-2.34), 0.16, 0.55 | **1.71 (1.09-2.67), 0.019*, 0.37** |
|  | CC | 40 | 20/9/11 | 1.53 (0.69-3.43), 0.30, 0.67 | 1.76 (0.86-3.59), 0.12, 0.52 |
|  | GC/CC | 194 | 94/46/54 | 1.45 (0.91-2.31), 0.12, 0.52 | **1.72 (1.13-2.62), 0.012*, 0.31** |
| *IL18* | GG | 138 | 54/38/46 |  |  |
| rs1946518 | GT | 190 | 89/50/51 | 1.53 (0.92-2.52), 0.099, 0.52 | 1.48 (0.93-2.33), 0.095, 0.52 |
|  | TT | 63 | 28/17/18 | 1.56 (0.78-3.11), 0.21, 0.58 | 1.25 (0.67-2.33), 0.49, 0.91 |
|  | GT/TT | 253 | 117/67/69 | 1.53 (0.95-2.46), 0.077, 0.49 | 1.42 (0.92-2.18), 0.12, 0.52 |
| *IL18* | AA | 191 | 73/58/60 |  |  |
| rs360719 | AG | 156 | 75/38/43 | 1.41 (0.86-2.31), 0.17, 0.55 | **1.60 (1.03-2.49), 0.037*, 0.40** |
|  | GG | 40 | 20/9/11 | 1.48 (0.66-3.33), 0.34, 0.72 | 1.64 (0.80-3.34), 0.18, 0.55 |
|  | AG/GG | 196 | 95/47/54 | 1.43 (0.90-2.27), 0.13, 0.54 | **1.61 (1.06-2.44), 0.026*, 0.39** |
| *JAK2* | TT | 180 | 80/41/59 |  |  |
| rs12343867 | TC | 174 | 76/51/47 | 1.42 (0.88-2.31), 0.15, 0.55 | 1.02 (0.66-1.57), 0.92, 0.99 |
|  | CC | 36 | 16/11/9 | 1.64 (0.70-3.83), 0.26, 0.65 | 1.04 (0.50-2.17), 0.91, 0.99 |
|  | TC/CC | 210 | 92/62/56 | 1.46 (0.92-2.31), 0.11, 0.52 | 1.03 (0.68-1.55), 0.91, 0.99 |
| *NLRP1* | AA | 113 | 48/31/34 |  |  |
| rs2670660 | AG | 195 | 92/43/60 | 0.96 (0.57-1.63), 0.89, 0.99 | 1.21 (0.75-1.96), 0.43, 0.83 |
|  | GG | 77 | 29/31/17 | 1.24 (0.61-2.49), 0.56, 0.99 | 0.73 (0.40-1.35), 0.32, 0.68 |
|  | AG/GG | 272 | 121/74/77 | 1.02 (0.62-1.70), 0.92, 0.99 | 1.06 (0.67-1.66), 0.81, 0.99 |
| *NLRP1* | GG | 110 | 46/31/33 |  |  |
| rs878329 | GC | 206 | 92/51/63 | 0.97 (0.57-1.65), 0.9, 0.99 | 1.10 (0.68-1.77), 0.71, 0.99 |
|  | CC | 70 | 29/24/17 | 1.08 (0.53-2.2), 0.84, 0.99 | 0.87 (0.46-1.63), 0.66, 0.99 |
|  | GC/CC | 276 | 121/75/80 | 0.99 (0.6-1.65), 0.98, 0.99 | 1.03 (0.65-1.64), 0.88, 0.99 |
| *NLRP3* | CC | 138 | 65/39/34 |  |  |
| rs10754558 | CG | 184 | 77/47/60 | **0.58 (0.34-0.98), 0.040*, 0.4** | 0.78 (0.50-1.24), 0.30, 0.67 |
|  | GG | 63 | 27/17/19 | 0.76 (0.38-1.53), 0.45, 0.86 | 0.86 (0.46-1.59), 0.62, 0.99 |
|  | CG/GG | 247 | 104/64/79 | 0.62 (0.38-1.02), 0.06, 0.48 | 0.80 (0.52-1.23), 0.32, 0.68 |
| *TBX21* | TT | 264 | 113/67/84 |  |  |
| rs17250932 | TC | 114 | 51/34/29 | 1.20 (0.72-2.02), 0.48, 0.91 | 1.04 (0.66-1.64), 0.86, 0.99 |
|  | CC | 6 | 3/2/1 | 3.43 (0.36-32.82), 0.29, 0.67 | 1.55 (0.29-8.31), 0.61, 0.99 |
|  | TC/CC | 120 | 54/36/30 | 1.27 (0.76-2.11), 0.36, 0.75 | 1.06 (0.68-1.65), 0.79, 0.99 |
| *TIRAP* | CC | 312 | 139/80/93 |  |  |
| rs8177374 | CT | 74 | 33/23/18 | 1.32 (0.72-2.43), 0.37, 0.75 | 0.99 (0.59-1.67), 0.98, 0.99 |
|  | TT | 4 | 0/1/3 | 0.11 (0.01-1.17), 0.068, 0.49 | - |
|  | CT/TT | 78 | 33/24/21 | 1.13 (0.63-2.02), 0.67, 0.99 | 0.90 (0.54-1.51), 0.70, 0.99 |
| ***TLR1*** | TT | 235 | 104/64/67 |  |  |
| **rs4833095** | TC | 136 | 55/41/40 | 1.11 (0.68-1.82), 0.68, 0.99 | 0.91 (0.58-1.41), 0.66, 0.99 |
|  | CC | 15 | 11/1/3 | 1.05 (0.28-3.96), 0.95, 0.99 | 2.93 (0.88-9.76), 0.079, 0.49 |
|  | TC/CC | 151 | 66/42/43 | 1.10 (0.68-1.78), 0.69, 0.99 | 1.02 (0.66-1.55), 0.95, 0.99 |
| *TLR5* | TT | 126 | 53/37/36 |  |  |
| rs5744174 | TC | 176 | 76/47/53 | 1.06 (0.62-1.80), 0.83, 0.99 | 1.15 (0.71-1.85), 0.58, 0.99 |
|  | CC | 86 | 41/20/25 | 1.09 (0.58-2.07), 0.78, 0.99 | 1.40 (0.80-2.47), 0.24, 0.63 |
|  | TC/CC | 262 | 117/67/78 | 1.07 (0.65-1.75), 0.79, 0.99 | 1.23 (0.79-1.91), 0.37, 0.75 |

| Logistic regression, adjusted for gender, HAQ-, DAS28-, DMARD at baseline. OR: odds ratio; EULAR, G/M/N: European League Against Rheumatism response criteria, good/moderate/none. Freq.: frequency. Correction for multiple testing using False Discovery Rate classical one-stage method set at 0.05 (q-value), based on 119 tests in IgM-rheumatoid factor stratified analyses. |
| --- |

**S3c Table. EULAR anti-TNF treatment response - seronegative RA patients.** Adjusted odds ratios for associations between genotypes and EULAR anti-TNF treatment response.

|  |  |  |  |  |  |
| --- | --- | --- | --- | --- | --- |
|  |  |  |  | G&M vs. N | G vs. M&N |
|  |  |  |  | Adjusted | Adjusted |
| Gene  (SNP) | Genotype | Frequency | G/M/N | OR (95% CI), p-, q-value | OR (95% CI), p-, q-value |
| ***TLR5*** | TT | 44 | 8/18/18 |  |  |
| **rs5744174** | TC | 58 | 28/15/15 | 1.97 (0.82-4.76), 0.13, 0.54 | 5.68 (2.07-15.55), 0.0007***, 0.042* |
|  | CC | 21 | 12/6/3 | 5.59 (1.29-24.32), 0.022*, 0.37 | 7.86 (2.25-27.39), 0.001**, 0.048* |
|  | TC/CC | 79 | 40/21/18 | 2.48 (1.07-5.72), 0.033*, 0.40 | 6.22 (2.37-16.29), 0.0002***, 0.024* |

Logistic regression, adjusted for gender, HAQ-, DAS28-, DMARD at baseline. OR: odds ratio; EULAR, G/M/N: European League Against Rheumatism response criteria, good/moderate/none. Freq.: frequency. Correction for multiple testing using False Discovery Rate classical one-stage method set at 0.05 (q-value), based on 119 tests in IgM-rheumatoid factor stratified analyses.
