# Supplementary material for: Genetic Variations in Pattern Recognition Receptor Loci Are Associated with Anti-TNF Response in Patients with Rheumatoid Arthritis
Source: PLoS One. 2015 Oct 6;10(10):e0139781. doi: 10.1371/journal.pone.0139781 (PMC4595012; doi:10.1371/journal.pone.0139781)
Supplement: S4 Table — Odds ratio for variant allele carriers association with EULAR good vs. moderate/none response. (a. EULAR good/moderate vs. non-response. b. EULAR good vs. moderate/non-response). (DOCX) [file pone.0139781.s005.docx]

**S4a Table. Anti-tumour necrosis factor (TNF) drug stratified analyses.** Odds ratio for variant allele carriers association with EULAR good/moderate vs. none response

|  |  | Infliximab (n=168) | Etanercept (n=166) | Adalimumab (n=134) | Monoclonal ab. (n=351) |
| --- | --- | --- | --- | --- | --- |
| SNP | Genotype | Adj. OR (95% CI), p-, q-value | Adj. OR (95% CI), p-, q-value | Adj. OR (95% CI), p-, q-value | Adj. OR (95% CI), p-, q-value |
| *CARD8*  rs2043211 | A/T | 0.94 (0.47-1.90), 0.87, 0.96 | 0.93 (0.45-1.93), 0.86, 0.96 | 1.53 (0.62-3.79), 0.36, 0.82 | 1.03 (0.62-1.70), 0.92, 0.97 |
| *IFNGR1*  rs2234711 | C/T | 0.84 (0.42-1.67), 0.61, 0.88 | 1.13 (0.54-2.35), 0.75, 0.94 | 0.98 (0.38-2.51), 0.97, 0.98 | 0.90 (0.54-1.51), 0.70, 0.91 |
| *IFNGR2* rs17882748 | T/C | 0.94 (0.41-2.16), 0.89, 0.96 | 0.81 (0.36-1.80), 0.60, 0.88 | 0.88 (0.28-2.78), 0.83, 0.96 | 0.80 (0.45-1.44), 0.47, 0.85 |
| *IFNGR2* rs8126756 | T/C | 1.48 (0.57-3.87), 0.42, 0.83 | 0.80 (0.34-1.92), 0.62, 0.88 | 1.08 (0.35-3.30), 0.89, 0.96 | 1.58 (0.81-3.05), 0.18, 0.67 |
| *IL12B* rs3212217 | G/C | 0.53 (0.26-1.09), 0.084, 0.66 | 0.97 (0.45-2.07), 0.94, 0.97 | 1.82 (0.67-4.90), 0.24, 0.73 | 0.79 (0.47-1.31), 0.36, 0.82 |
| ***IL12B* rs3212227** | A/C | 0.51 (0.25-1.06), 0.07, 0.66 | 1.02 (0.48-2.16), 0.96, 0.98 | 2.37 (0.79-7.08), 0.12, 0.66 | 0.84 (0.50-1.43), 0.53, 0.86 |
| ***IL12B* rs6887695** | G/C | 0.52 (0.26-1.04), 0.066, 0.66 | 1.04 (0.50-2.17), 0.91, 0.96 | 0.43 (0.16-1.12), 0.085, 0.66 | **0.47 (0.28-0.79), 0.004**, 0.61** |
| *IL12RB1* rs401502 | C/G | 1.48 (0.74-2.98), 0.27, 0.78 | 0.60 (0.29-1.26), 0.18, 0.67 | 1.81 (0.71-4.63), 0.22, 0.72 | 1.43 (0.86-2.37), 0.17, 0.67 |
| *IL12RB2* rs11810249 | C/T | Not enough variants for analysis | Not enough variants for analysis | Not enough variants for analysis | Not enough variants for analysis |
| *IL18*  rs187238 | G/C | 1.38 (0.69-2.76), 0.36, 0.82 | 1.37 (0.67-2.80), 0.39, 0.83 | 1.38 (0.55-3.46), 0.50, 0.86 | 1.49 (0.90-2.47), 0.12, 0.66 |
| *IL18* rs1946518 | G/T | 1.56 (0.79-3.08), 0.20, 0.72 | 1.47 (0.71-3.06), 0.30, 0.82 | 1.18 (0.43-3.22), 0.74, 0.94 | 1.54 (0.92-2.58), 0.098, 0.66 |
| *IL18*  rs360719 | A/G | 1.26 (0.63-2.51), 0.52, 0.86 | 1.35 (0.65-2.77), 0.42, 0.83 | 1.39 (0.56-3.46), 0.48, 0.86 | 1.41 (0.86-2.33), 0.18, 0.67 |
| *JAK2* rs12343867 | T/C | 1.59 (0.78-3.24), 0.20, 0.72 | 1.62 (0.79-3.30), 0.19, 0.70 | 2.16 (0.84-5.50), 0.11, 0.66 | 1.53 (0.93-2.54), 0.096, 0.66 |
| ***NLRP1* rs2670660** | A/G | 1.82 (0.87-3.81), 0.11, 0.66 | 1.23 (0.56-2.70), 0.61, 0.88 | 1.30 (0.48-3.56), 0.61, 0.88 | 1.25 (0.73-2.14), 0.41, 0.83 |
| ***NLRP1* rs878329** | G/C | **2.54 (1.20-5.38), 0.015*, 0.66** | 0.76 (0.33-1.74), 0.52, 0.86 | 1.55 (0.59-4.10), 0.38, 0.83 | 1.62 (0.95-2.76), 0.074, 0.66 |
| *NLRP3* rs10754558 | C/G | 0.59 (0.29-1.19), 0.14, 0.66 | 0.51 (0.22-1.20), 0.12, 0.66 | 0.52 (0.18-1.50), 0.22, 0.72 | 0.71 (0.42-1.21), 0.21, 0.72 |
| *TBX21* rs17250932 | T/C | 1.07 (0.51-2.24), 0.86, 0.96 | 1.08 (0.50-2.34), 0.85, 0.96 | 1.28 (0.45-3.66), 0.65, 0.91 | 1.17 (0.67-2.04), 0.57, 0.88 |
| *TIRAP* rs8177374 | C/T | 0.78 (0.33-1.85), 0.57, 0.88 | 1.43 (0.59-3.44), 0.43, 0.83 | 2.63 (0.69-9.97), 0.16, 0.67 | 1.36 (0.70-2.64), 0.36, 0.82 |
| *TLR1* rs4833095 | T/C | 0.80 (0.40-1.60), 0.52, 0.86 | 0.95 (0.46-1.97), 0.89, 0.96 | 2.15 (0.75-6.19), 0.16, 0.67 | 1.19 (0.70-2.01), 0.53, 0.86 |
| *TLR5* rs5744174 | T/C | 1.56 (0.76-3.19), 0.22, 0.72 | 1.57 (0.74-3.36), 0.24, 0.73 | 0.61 (0.21-1.75), 0.36, 0.82 | 1.08 (0.63-1.84), 0.77, 0.95 |

Logistic regression, adjusted for gender, HAQ-, DAS28-, and DMARD at baseline. Patients treated with certolizumab and golimumab were too few for analysis. Adj. OR: adjusted odds ratio. Monoclonal antibodies: infliximab, adalimumab, and golimumab. Freq.: frequency. Correction for multiple testing using False Discovery Rate classical one-stage method set at 0.05 (q-value), based on 152 tests in drug stratified analyses.

**S4b Table.** **Anti-tumour necrosis factor (TNF) drug stratified analyses.** Odds ratio for variant allele carriers association with EULAR good vs. moderate/none response

|  |  | Infliximab (n=168) | Etanercept (n=166) | Adalimumab (n=134) | Monoclonal antibodies (n=351) |
| --- | --- | --- | --- | --- | --- |
| SNP | Genotype | OR (95% CI), p-, q-value | OR (95% CI), p-, q-value | OR (95% CI), p-, q-value | OR (95% CI), p-, q-value |
| *CARD8*  rs2043211 | A/T | 1.77 (0.83-3.76), 0.14, 0.66 | 1.39 (0.72-2.69), 0.32, 0.82 | 1.02 (0.50-2.06), 0.97, 0.98 | 1.32 (0.83-2.08), 0.24, 0.73 |
| *IFNGR1*  rs2234711 | C/T | 0.72 (0.36-1.45), 0.35, 0.82 | 1.10 (0.56-2.15), 0.78, 0.95 | 1.85 (0.89-3.84), 0.099, 0.66 | 1.15 (0.73-1.82), 0.54, 0.86 |
| *IFNGR2* rs17882748 | T/C | 1.10 (0.46-2.61), 0.83, 0.96 | 1.38 (0.67-2.83), 0.38, 0.83 | 0.62 (0.27-1.45), 0.27, 0.78 | 0.70 (0.42-1.17), 0.18, 0.67 |
| *IFNGR2* rs8126756 | T/C | 1.38 (0.55-3.46), 0.50, 0.86 | 1.08 (0.48-2.43), 0.86, 0.96 | 1.14 (0.50-2.62), 0.75, 0.94 | 1.55 (0.89-2.71), 0.12, 0.66 |
| *IL12B* rs3212217 | G/C | 0.51 (0.23-1.13), 0.096, 0.66 | 1.15 (0.58-2.26), 0.69, 0.91 | 1.44 (0.70-2.96), 0.32, 0.82 | 0.85 (0.53-1.36), 0.51, 0.86 |
| *IL12B* rs3212227 | A/C | 0.53 (0.24-1.19), 0.12, 0.66 | 1.15 (0.59-2.25), 0.68, 0.91 | 1.54 (0.73-3.23), 0.26, 0.77 | 0.90 (0.56-1.45), 0.67, 0.91 |
| *IL12B* rs6887695 | G/C | 0.77 (0.38-1.55), 0.46, 0.85 | 1.07 (0.56-2.03), 0.84, 0.96 | 0.72 (0.35-1.46), 0.36, 0.82 | 0.69 (0.44-1.08), 0.10, 0.66 |
| *IL12RB1* rs401502 | C/G | 0.93 (0.46-1.89), 0.85, 0.96 | 0.61 (0.32-1.17), 0.14, 0.66 | 2.08 (1.00-4.35), 0.051, 0.66 | 1.46 (0.93-2.29), 0.10, 0.66 |
| *IL12RB2* rs11810249 | C/T | Not enough variants for analysis | Not enough variants for analysis | Not enough variants for analysis | Not enough variants for analysis |
| *IL18*  rs187238 | G/C | 1.39 (0.68-2.86), 0.37, 0.83 | 1.31 (0.69-2.49), 0.41, 0.83 | 1.09 (0.53-2.24), 0.82, 0.96 | 1.51 (0.96-2.38), 0.077, 0.66 |
| *IL18* rs1946518 | G/T | 0.99 (0.49-2.02), 0.99, 0.99 | 1.23 (0.63-2.38), 0.55, 0.86 | 0.88 (0.40-1.92), 0.75, 0.94 | 1.21 (0.76-1.94), 0.42, 0.83 |
| *IL18*  rs360719 | A/G | 1.17 (0.58-2.37), 0.66, 0.91 | 1.21 (0.63-2.32), 0.57, 0.88 | 1.16 (0.57-2.36), 0.69, 0.91 | 1.43 (0.91-2.24), 0.12, 0.66 |
| *JAK2* rs12343867 | T/C | 1.05 (0.51-2.16), 0.89, 0.96 | 1.02 (0.54-1.92), 0.95, 0.97 | 1.28 (0.63-2.58), 0.50, 0.86 | 0.95 (0.61-1.49), 0.82, 0.96 |
| *NLRP1* rs2670660 | A/G | 1.03 (0.47-2.27), 0.94, 0.97 | 1.33 (0.64-2.74), 0.44, 0.85 | 1.33 (0.62-2.85), 0.47, 0.85 | 0.92 (0.57-1.50), 0.75, 0.94 |
| *NLRP1* rs878329 | G/C | 1.47 (0.65-3.31), 0.35, 0.82 | 0.86 (0.41-1.79), 0.68, 0.91 | 1.51 (0.71-3.24), 0.29, 0.81 | 1.14 (0.70-1.87), 0.59, 0.88 |
| ***NLRP3* rs10754558** | C/G | **0.46 (0.22-0.94), 0.032*, 0.66** | 0.72 (0.35-1.46), 0.36, 0.82 | 0.73 (0.34-1.57), 0.42, 0.83 | 0.70 (0.44-1.11), 0.13, 0.66 |
| *TBX21* rs17250932 | T/C | 0.95 (0.44-2.02), 0.89, 0.96 | 1.03 (0.52-2.06), 0.93, 0.97 | 1.31 (0.59-2.90), 0.50, 0.86 | 0.94 (0.58-1.54), 0.82, 0.96 |
| *TIRAP* rs8177374 | C/T | 1.16 (0.48-2.80), 0.75, 0.94 | 0.81 (0.38-1.74), 0.60, 0.88 | 0.50 (0.21-1.19), 0.12, 0.66 | 0.80 (0.45-1.43), 0.46, 0.85 |
| *TLR1* rs4833095 | T/C | 0.68 (0.32-1.42), 0.30, 0.82 | 1.68 (0.87-3.24), 0.12, 0.66 | 0.94 (0.44-1.99), 0.87, 0.96 | 0.91 (0.57-1.45), 0.68, 0.91 |
| ***TLR5* rs5744174** | T/C | **2.65 (1.18-5.91), 0.018*, 0.66** | **2.34 (1.13-4.84), 0.022*, 0.66** | 0.82 (0.38-1.74), 0.60, 0.88 | 1.4 (0.87-2.26), 0.17, 0.67 |

Logistic regression, adjusted for gender, HAQ-, DAS28-, and DMARD at baseline. Patients treated with certolizumab and golimumab were too few for analysis. OR: odds ratio. Monoclonal antibodies: infliximab, adalimumab, and golimumab. Freq.: frequency. Correction for multiple testing using False Discovery Rate classical one-stage method set at 0.05 (q-value), based on 152 tests in drug stratified analyses.
