# Supplementary material for: Genetic Variations in Pattern Recognition Receptor Loci Are Associated with Anti-TNF Response in Patients with Rheumatoid Arthritis
Source: PLoS One. 2015 Oct 6;10(10):e0139781. doi: 10.1371/journal.pone.0139781 (PMC4595012; doi:10.1371/journal.pone.0139781)
Supplement: S5 Table — All RA patients. Adjusted odds ratios for associations between genotypes and EULAR anti-TNF treatment response. (DOCX) [file pone.0139781.s006.docx]

**S5 Table. EULAR anti-TNF treatment response at 16 weeks.** All RA patients. Adjusted odds ratios for associations between genotypes and EULAR anti-TNF treatment response.

|  |  |  |  |  |  |
| --- | --- | --- | --- | --- | --- |
|  |  |  |  | G&M vs. N | G vs. M&N |
|  |  |  |  | Adjusted | Adjusted |
| *Gene*  SNP | Geno-type | Freq. | G/M/N | OR (95% CI), p-, q-value | OR (95% CI), p-, q-value |
| *CARD8* | AA | 159 | 58/51/50 |  |  |
| rs2043211 | AT | 174 | 77/53/44 | 1.41 (0.85-2.35), 0.18, 0.60 | 1.39 (0.89-2.18), 0.15, 0.56 |
|  | TT | 44 | 15/9/20 | 0.5 (0.24-1.02), 0.055, 0.47 | 0.86 (0.42-1.76), 0.67, 0.80 |
|  | AT/TT | 218 | 92/62/64 | 1.11 (0.69-1.77), 0.67, 0.80 | 1.26 (0.82-1.94), 0.29, 0.66 |
| *IFNGR1* | TT | 154 | 59/51/44 |  |  |
| rs2234711 | TC | 174 | 72/52/50 | 1.02 (0.61-1.68), 0.95, 0.96 | 1.16 (0.74-1.83), 0.51, 0.72 |
|  | CC | 55 | 22/13/20 | 0.75 (0.38-1.49), 0.42, 0.68 | 1.12 (0.58-2.13), 0.74, 0.82 |
|  | TC/CC | 229 | 94/65/70 | 0.94 (0.59-1.51), 0.80, 0.86 | 1.15 (0.75-1.77), 0.52, 0.72 |
| *IFNGR2* | CC | 103 | 47/31/25 |  |  |
| rs17882748 | CT | 179 | 68/48/63 | 0.59 (0.33-1.05), 0.074, 0.47 | 0.77 (0.46-1.27), 0.30, 0.66 |
|  | TT | 89 | 35/28/26 | 0.65 (0.33-1.27), 0.21, 0.63 | 0.76 (0.42-1.38), 0.37, 0.68 |
|  | CT/TT | 268 | 103/76/89 | 0.61 (0.35-1.05), 0.075, 0.47 | 0.77 (0.48-1.22), 0.27, 0.66 |
| *IFNGR2* | TT | 303 | 116/94/93 |  |  |
| rs8126756 | TC | 63 | 31/17/15 | 1.97 (0.99-3.93), 0.055, 0.47 | 1.73 (0.98-3.04), 0.057, 0.47 |
|  | CC | 8 | 3/2/3 | 0.53 (0.11-2.53), 0.43, 0.68 | 0.88 (0.20-3.97), 0.87, 0.90 |
|  | TC/CC | 71 | 34/19/18 | 1.67 (0.88-3.18), 0.12, 0.56 | 1.60 (0.94-2.74), 0.085, 0.49 |
| *IL12B* | GG | 240 | 98/73/69 |  |  |
| rs3212217 | GC | 121 | 45/37/39 | 0.81 (0.49-1.34), 0.41, 0.68 | 0.81 (0.51-1.28), 0.37, 0.68 |
|  | CC | 18 | 8/5/5 | 1.21 (0.38-3.82), 0.75, 0.82 | 1.15 (0.42-3.14), 0.78, 0.85 |
|  | GC/CC | 139 | 53/42/44 | 0.85 (0.53-1.38), 0.52, 0.72 | 0.85 (0.55-1.31), 0.46, 0.69 |
| *IL12B* | AA | 242 | 99/73/70 |  |  |
| rs3212227 | AC | 118 | 45/37/36 | 0.85 (0.51-1.42), 0.54, 0.72 | 0.83 (0.52-1.31), 0.42, 0.68 |
|  | CC | 18 | 8/5/5 | 1.21 (0.38-3.81), 0.75, 0.82 | 1.14 (0.42-3.09), 0.80, 0.86 |
|  | AC/CC | 136 | 53/42/41 | 0.89 (0.55-1.45), 0.64, 0.79 | 0.86 (0.55-1.34), 0.51, 0.72 |
| ***IL12B*** | GG | 184 | 78/58/48 |  |  |
| **rs6887695** | GC | 163 | 65/41/57 | **0.58 (0.35-0.95), 0.029*, 0.47** | 0.87 (0.56-1.34), 0.52, 0.72 |
|  | CC | 37 | 12/17/8 | 1.18 (0.48-2.90), 0.71, 0.82 | 0.67 (0.31-1.44), 0.31, 0.66 |
|  | GC/CC | 200 | 77/58/65 | 0.65 (0.41-1.04), 0.075, 0.47 | 0.83 (0.54-1.26), 0.38, 0.68 |
| *IL12RB1* | CC | 185 | 76/57/52 |  |  |
| rs401502 | CG | 162 | 69/47/46 | 0.86 (0.53-1.42), 0.57, 0.73 | 0.96 (0.61-1.48), 0.84, 0.88 |
|  | GG | 32 | 11/9/12 | 0.70 (0.30-1.61), 0.40, 0.68 | 0.67 (0.30-1.51), 0.34, 0.67 |
|  | CG/GG | 194 | 80/56/58 | 0.83 (0.52-1.34), 0.45, 0.69 | 0.90 (0.59-1.38), 0.64, 0.79 |
| *IL12RB2* | CC | 385 | 155/116/114 |  |  |
| rs11810249 | CT | 0 | 0/0/0 | Not enough variant for analyses | Not enough variant for analyses |
|  | TT | 0 | 0/0/0 |  |  |
|  | CT/TT | 0 | 0/0/0 |  |  |
| *IL18* | GG | 182 | 68/55/59 |  |  |
| rs187238 | GC | 156 | 69/43/44 | 1.50 (0.91-2.47), 0.11, 0.56 | 1.51 (0.96-2.37), 0.074, 0.47 |
|  | CC | 41 | 14/15/12 | 1.52 (0.69-3.34), 0.29, 0.66 | 1.03 (0.49-2.14), 0.94, 0.96 |
|  | GC/CC | 197 | 83/58/56 | 1.51 (0.94-2.41), 0.087, 0.49 | 1.40 (0.91-2.15), 0.12, 0.56 |
| *IL18* | GG | 137 | 51/40/46 |  |  |
| rs1946518 | GT | 185 | 84/52/49 | **1.71 (1.02-2.87), 0.041*, 0.47** | 1.56 (0.98-2.49), 0.061, 0.47 |
|  | TT | 61 | 20/22/19 | 1.48 (0.74-2.97), 0.26, 0.66 | 0.93 (0.48-1.79), 0.82, 0.87 |
|  | GT/TT | 246 | 104/74/68 | **1.65 (1.02-2.68), 0.042*, 0.47** | 1.38 (0.89-2.16), 0.15, 0.56 |
| *IL18* | AA | 183 | 69/56/58 |  |  |
| rs360719 | AG | 157 | 69/44/44 | 1.45 (0.88-2.38), 0.15, 0.56 | 1.44 (0.92-2.26), 0.11, 0.56 |
|  | GG | 41 | 14/15/12 | 1.46 (0.66-3.22), 0.35, 0.68 | 0.99 (0.47-2.06), 0.97, 0.97 |
|  | AG/GG | 198 | 83/59/56 | 1.45 (0.90-2.32), 0.12, 0.56 | 1.34 (0.88-2.05), 0.18, 0.60 |
| *JAK2* | TT | 185 | 74/48/63 |  |  |
| rs12343867 | TC | 169 | 70/55/44 | **1.78 (1.09-2.90), 0.022*, 0.47** | 1.17 (0.75-1.80), 0.49, 0.72 |
|  | CC | 31 | 12/12/7 | 1.80 (0.71-4.56), 0.21, 0.63 | 0.97 (0.44-2.15), 0.94, 0.96 |
|  | TC/CC | 200 | 82/67/51 | **1.78 (1.11-2.85), 0.016*, 0.47** | 1.13 (0.74-1.72), 0.56, 0.73 |
| *NLRP1* | AA | 113 | 42/32/39 |  |  |
| rs2670660 | AG | 180 | 81/48/51 | 1.33 (0.78-2.27), 0.29, 0.66 | 1.39 (0.85-2.28), 0.19, 0.61 |
|  | GG | 87 | 30/36/21 | 1.34 (0.69-2.59), 0.38, 0.68 | 0.78 (0.43-1.43), 0.43, 0.68 |
|  | AG/GG | 267 | 111/84/72 | 1.33 (0.81-2.20), 0.26, 0.66 | 1.16 (0.73-1.84), 0.53, 0.72 |
| *NLRP1* | GG | 109 | 41/30/38 |  |  |
| rs878329 | GC | 192 | 84/56/52 | 1.50 (0.88-2.56), 0.14, 0.56 | 1.27 (0.78-2.08), 0.34, 0.67 |
|  | CC | 81 | 28/30/23 | 1.13 (0.58-2.18), 0.72, 0.82 | 0.78 (0.42-1.44), 0.43, 0.68 |
|  | GC/CC | 273 | 112/86/75 | 1.38 (0.83-2.27), 0.21, 0.63 | 1.10 (0.69-1.76), 0.68, 0.80 |
| *NLRP3* | CC | 138 | 59/42/37 |  |  |
| rs10754558 | CG | 176 | 69/51/56 | 0.68 (0.40-1.14), 0.14, 0.56 | 0.84 (0.53-1.34), 0.47, 0.70 |
|  | GG | 65 | 25/20/20 | 0.81 (0.41-1.61), 0.55, 0.73 | 0.85 (0.46-1.58), 0.60, 0.76 |
|  | CG/GG | 241 | 94/71/76 | 0.71 (0.43-1.16), 0.18, 0.60 | 0.84 (0.55-1.31), 0.45, 0.69 |
| *TBX21* | TT | 253 | 103/69/81 |  |  |
| rs17250932 | TC | 114 | 44/41/29 | 1.30 (0.77-2.19), 0.32, 0.67 | 0.90 (0.57-1.43), 0.66, 0.80 |
|  | CC | 6 | 3/2/1 | 2.89 (0.31-27.26), 0.36, 0.68 | 1.64 (0.31-8.78), 0.57, 0.73 |
|  | TC/CC | 120 | 47/43/30 | 1.35 (0.81-2.26), 0.25, 0.66 | 0.93 (0.59-1.46), 0.74, 0.82 |
| *TIRAP* | CC | 302 | 126/84/92 |  |  |
| rs8177374 | CT | 76 | 28/30/18 | 1.27 (0.69-2.34), 0.44, 0.68 | 0.74 (0.43-1.26), 0.26, 0.66 |
|  | TT | 4 | 0/1/3 | 0.12 (0.01-1.21), 0.072, 0.47 | - |
|  | CT/TT | 80 | 28/31/21 | 1.10 (0.62-1.97), 0.75, 0.82 | 0.68 (0.40-1.16), 0.15, 0.56 |
| ***TLR1*** | TT | 237 | 93/73/71 |  |  |
| **rs4833095** | TC | 129 | 52/41/36 | 1.28 (0.77-2.13), 0.34, 0.67 | 1.12 (0.71-1.76), 0.62, 0.78 |
|  | CC | 16 | 11/2/3 | 1.74 (0.45-6.71), 0.42, 0.68 | **3.63 (1.18-11.18), 0.025*, 0.47** |
|  | TC/CC | 145 | 63/43/39 | 1.32 (0.81-2.16), 0.27, 0.66 | 1.27 (0.83-1.96), 0.28, 0.66 |
| ***TLR5*** | TT | 123 | 42/41/40 |  |  |
| **rs5744174** | TC | 178 | 76/51/51 | 1.30 (0.77-2.21), 0.33, 0.67 | 1.60 (0.98-2.62), 0.059, 0.47 |
|  | CC | 77 | 34/21/22 | 1.43 (0.74-2.78), 0.29, 0.66 | 1.76 (0.97-3.22), 0.065, 0.47 |
|  | TC/CC | 255 | 110/72/73 | 1.34 (0.81-2.20), 0.25, 0.66 | **1.65 (1.04-2.62), 0.034*, 0.47** |

| Logistic regression, adjusted for gender, HAQ-, DAS28-, DMARD at baseline. OR: odds ratio; EULAR, G/M/N: European League Against Rheumatism response criteria, good/moderate/none. Freq.: frequency. Correction for multiple testing using False Discovery Rate classical one-stage method set at 0.05 (q-value), based on 113 tests in analyses of primary outcome. |
| --- |
