# Supplementary material for: Genetic Variations in Pattern Recognition Receptor Loci Are Associated with Anti-TNF Response in Patients with Rheumatoid Arthritis
Source: PLoS One. 2015 Oct 6;10(10):e0139781. doi: 10.1371/journal.pone.0139781 (PMC4595012; doi:10.1371/journal.pone.0139781)
Supplement: S6 Table — Multivariate logistic regression. Odds ratio (OR) per variable in model for European League Against Rheumatism response criteria, good/moderate/none (EULAR, G/M/N). Seropositive/-negative RA: rheumatoid arthritis positive/negative for IgM-rheumatoid factor. DAS28: disease activity score across 28-joints. HAQ: health assessment questionnaire score. DMARD: disease-modifying anti-rheumatic drugs (dichotome). Smoking: previous/current smoking (dichotome). Erosions: erosions on x-ray (dichotome). TJC: tender joint count. VAS: visual analogue scale. P-value: * <0.05. ** <0.01. *** <0.001. (DOCX) [file pone.0139781.s007.docx]

**Table S6. IgM-** **Rheumatoid factor stratified analyses adjusted for potential confounders.**

Multivariate logistic regression. Odds ratio (OR) per variable in model for European League Against Rheumatism response criteria, good/moderate/none (EULAR, G/M/N). Seropositive/-negative RA: rheumatoid arthritis positive/negative for IgM-rheumatoid factor. DAS28: disease activity score across 28-joints. HAQ: health assessment questionnaire score. DMARD: disease-modifying anti-rheumatic drugs (dichotome). Smoking: previous/current smoking (dichotome). Erosions: erosions on x-ray (dichotome). TJC: tender joint count. VAS: visual analogue scale. P-value: * <0.05. ** <0.01. *** <0.001

|  |  |
| --- | --- |
| EULAR G vs. MN | Seropositive RA |
| rs2043211_CARD_8 | OR (95% CI), p-value |
| AT | 1.76 (1.03-3.00), 0.037* |
| TT | 1.52 (0.66-3.48), 0.32 |
| DAS28 | 2.09 (1.35-3.23), 0.0009*** |
| HAQ | 0.40 (0.24-0.65), 0.0002*** |
| DMARD | 1.54 (0.74-3.21), 0.25 |
| male gender | 0.93 (0.51-1.69), 0.82 |
| smoking | 0.67 (0.38-1.18), 0.16 |
| erosions | 1.04 (0.59-1.83), 0.89 |
| age | 1.02 (1.00-1.05), 0.030* |
| TJC | 0.91 (0.85-0.97), 0.006** |
| pain VAS | 1.01 (0.99-1.03), 0.45 |
| patient global VAS | 0.99 (0.97-1.01), 0.42 |
| constant | 0.03 (0.00-0.27), 0.001** |
|  |  |
| EULAR GM vs. N | Seropositive RA |
| rs17882748_IFNGR2 | OR (95% CI), p-value |
| CT | 0.70 (0.34-1.44), 0.33 |
| TT | 0.40 (0.18-0.90), 0.027* |
| DAS28 | 2.92 (1.83-4.64), 0.00001*** |
| HAQ | 0.36 (0.22-0.61), 0.0001*** |
| DMARD | 1.92 (0.90-4.11), 0.09 |
| male gender | 1.29 (0.66-2.49), 0.46 |
| smoking | 0.95 (0.50-1.78), 0.87 |
| erosions | 0.90 (0.47-1.70), 0.74 |
| age | 1.01 (0.99-1.03), 0.39 |
| TJC | 0.93 (0.87-1.00), 0.045* |
| pain VAS | 1.01 (0.99-1.03), 0.51 |
| patient global VAS | 0.98 (0.96-1.01), 0.17 |
| constant | 0.08 (0.01-0.68), 0.021* |

|  |  |
| --- | --- |
| EULAR GM vs. N | Seropositive RA |
| rs6887695_IL12B | OR (95% CI), p-value |
| GC | 0.64 (0.36-1.15), 0.14 |
| CC | 0.96 (0.35-2.64), 0.93 |
| DAS28 | 2.93 (1.84-4.67), 0.00001*** |
| HAQ | 0.36 (0.21-0.60), 0.0001*** |
| DMARD | 2.05 (0.97-4.34), 0.06 |
| male gender | 1.30 (0.68-2.51), 0.43 |
| smoking | 0.85 (0.46-1.58), 0.61 |
| erosions | 0.82 (0.43-1.56), 0.55 |
| age | 1.01 (0.99-1.04), 0.24 |
| TJC | 0.92 (0.86-0.99), 0.018* |
| pain VAS | 1.01 (0.99-1.03), 0.39 |
| patient global VAS | 0.99 (0.96-1.01), 0.25 |
| constant | 0.05 (0.01-0.44), 0.007** |
|  |  |
| EULAR G vs. MN | Seropositive RA |
| rs187238_IL18 | OR (95% CI), p-value |
| GC | 1.73 (1.01-2.94), 0.045* |
| CC | 1.6 (0.62-4.15), 0.33 |
| DAS28 | 2.14 (1.39-3.31), 0.0006*** |
| HAQ | 0.40 (0.25-0.65), 0.0002*** |
| DMARD | 1.72 (0.82-3.62), 0.15 |
| male gender | 0.99 (0.54-1.8), 0.97 |
| smoking | 0.60 (0.34-1.06), 0.08 |
| erosions | 1.01 (0.57-1.80), 0.97 |
| age | 1.02 (1.00-1.05), 0.022* |
| TJC | 0.91 (0.86-0.98), 0.009** |
| pain VAS | 1.01 (0.99-1.03), 0.43 |
| patient global VAS | 0.99 (0.97-1.01), 0.38 |
| constant | 0.03 (0.00-0.22), 0.0007*** |

|  |  |
| --- | --- |
| EULAR G vs. MN | Seropositive RA |
| rs360719_IL18 | OR (95% CI), p-value |
| AG | 1.65 (0.97-2.80), 0.06 |
| GG | 1.51 (0.58-3.92), 0.40 |
| DAS28 | 2.11 (1.37-3.25), 0.0007*** |
| HAQ | 0.39 (0.24-0.63), 0.0001*** |
| DMARD | 1.62 (0.77-3.43), 0.20 |
| male gender | 0.87 (0.48-1.58), 0.66 |
| smoking | 0.58 (0.33-1.02), 0.06 |
| erosions | 1.06 (0.60-1.87), 0.84 |
| age | 1.02 (1.00-1.04), 0.046* |
| TJC | 0.92 (0.86-0.98), 0.010** |
| pain VAS | 1.01 (0.99-1.03), 0.30 |
| patient global VAS | 0.99 (0.97-1.01), 0.28 |
| constant | 0.04 (0.01-0.32), 0.002** |
|  |  |
| EULAR GM vs. N | Seropositive RA |
| rs10754558_NLRP3 | OR (95% CI), p-value |
| CG | 0.50 (0.26-0.94), 0.030* |
| GG | 0.53 (0.24-1.19), 0.12 |
| DAS28 | 2.67 (1.66-4.29), 0.00005*** |
| HAQ | 0.36 (0.21-0.60), 0.00008*** |
| DMARD | 2.03 (0.94-4.38), 0.07 |
| male gender | 1.43 (0.73-2.79), 0.30 |
| smoking | 0.87 (0.46-1.62), 0.65 |
| erosions | 0.86 (0.45-1.64), 0.66 |
| age | 1.01 (0.99-1.04), 0.31 |
| TJC | 0.94 (0.87-1.01), 0.08 |
| pain VAS | 1.01 (0.99-1.03), 0.37 |
| patient global VAS | 0.99 (0.96-1.01), 0.26 |
| constant | 0.08 (0.01-0.69), 0.022* |

|  |  |
| --- | --- |
| EULAR G vs. MN | Seronegative RA |
| rs5744174_TLR5 | OR (95% CI), p-value |
| TC | 4.03 (1.20-13.57), 0.024* |
| CC | 4.65 (1.12-19.38), 0.035* |
| DAS28 | 0.76 (0.32-1.78), 0.53 |
| HAQ | 0.54 (0.23-1.27), 0.16 |
| DMARD | 1.58 (0.31-8.20), 0.58 |
| male gender | 1.21 (0.34-4.25), 0.77 |
| smoking | 0.79 (0.28-2.19), 0.65 |
| erosions | 1.03 (0.36-2.89), 0.96 |
| age | 0.99 (0.95-1.02), 0.47 |
| TJC | 1.01 (0.91-1.11), 0.90 |
| pain VAS | 0.98 (0.94-1.03), 0.47 |
| patient global VAS | 1.02 (0.97-1.08), 0.41 |
| constant | 1.67 (0.04-66.52), 0.79 |
